# Multicycle *operando* pressure measurements enable assessment of redox mediator efficacy in lithium–oxygen batteries†

**DOI:** 10.1039/d5sc02350e

**Published:** 2025-05-20

**Authors:** Thukshan Samarakoon, Ben Wood, Alex R. Neale, Elliot Coulbeck, Daniel J. Saccomando, Laurence J. Hardwick

**Affiliations:** a Stephenson Institute for Renewable Energy, Department of Chemistry, University of Liverpool Liverpool L69 7ZF UK hardwick@liverpool.ac.uk; b Lubrizol Limited Blackley Manchester M9 8ES UK; c Lubrizol Limited Hazelwood Derby DE56 4AN UK

## Abstract

Redox mediators (RMs) present a promising strategy for achieving low overpotential charging of lithium–oxygen (Li–O_2_) batteries, thereby extending cycle life and improving overall energy efficiency. In this study, multi-cycle *operando* pressure measurement during galvanostatic Li–O_2_ cell cycling was employed to assess the efficacy of 2,2,6,6-tetramethylpiperdinyloxyl (TEMPO) as a charge RM in sulfolane- and diglyme-based electrolytes. In both mediated electrolytes, electrochemical TEMPO oxidation coincided with gas evolution, validating TEMPO activity and revealing distinct behaviour in the reactions and stability of the glyme- and sulfone-based electrolytes. Pressure measurements showed a greater extent of parasitic reactions during charging in the mediated diglyme system during early cycles. In the sulfolane-based electrolyte, initial stable cycling was observed. However, a more rapid capacity fade was subsequently observed in the latter cycles, due to increasing parasitic chemistry on charge. Furthermore, highly sensitive pressure measurements enabled small changes in the pressure response to be correlated with transitions in the electrochemical cycling profile. Analyses of the dynamic rate of pressure changes within Li–O_2_ cells and correlation with differential capacity was used to identify exact points within a charge step wherein RM efficacy is diminished, thereby tracking the evolution of RM activity loss during cycling. This approach provided a valuable indicator of RM efficacy, defined in terms of maximising the number of cycles for which gas evolution is centred around the RM oxidation potential. Importantly, this method directly assesses RM cyclability in the Li–O_2_ cell environment and can be applied to any electrolyte–electrode combination, proving to be a versatile approach for identification of promising mediated electrolyte formulations for longer life Li–O_2_ batteries.

## Abstract 2

Redox mediator (RM) efficacy during Li–O_2_ cell charging is maximised when gas evolution is centred around the RM oxidation potential.

## Graphical abstract

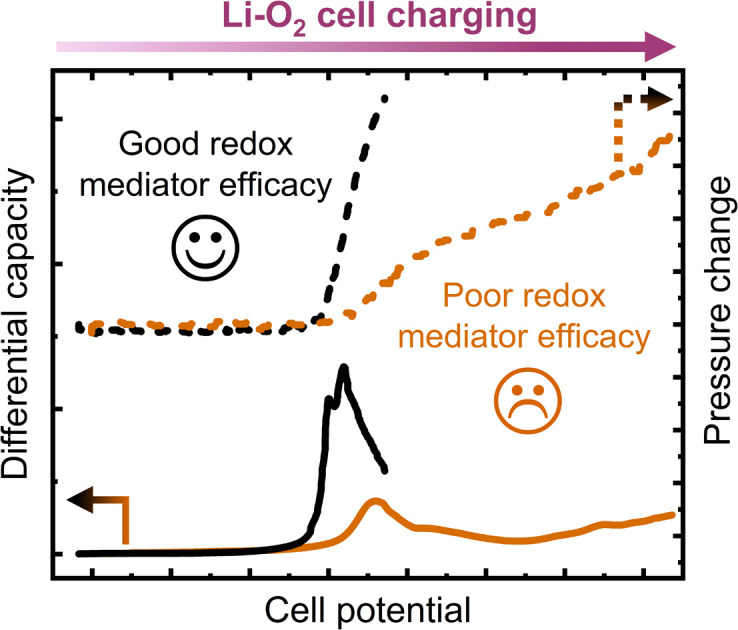

## Introduction

Lithium–oxygen (Li–O_2_) batteries offer a significantly higher theoretical specific energy (*ca.* 3500 Wh kg^−1^ based on lithium peroxide (Li_2_O_2_) formation/decomposition) compared to other battery chemistries.^1–3^ The electrochemical reactions taking place at the positive electrode are 2-electron oxygen reduction and evolution reactions (ORR and OER), forming and oxidatively decomposing Li_2_O_2_ on discharge and charge, respectively.^4–8^ Key mechanistic studies on ORR and OER in Li–O_2_ cells have developed the research community's understanding of factors governing the discharge mechanism (surface- *versus* solution-driven Li_2_O_2_ formation) and the associated Li_2_O_2_ morphology on the positive electrode,^4,9–14^ as well as charging mechanisms.^9,15,16^ The insulating nature of Li_2_O_2_ and distribution of the product on the electrode surface necessitates high overpotentials for its electrochemical oxidation on charge, driving high potential-induced parasitic reactions that irreversibly breakdown most organic electrolyte solvents and the carbon-based positive electrode.^17–19^ Ultimately, this leads to poor cycle life and low overall energy efficiency, diminishing the practical viability of Li–O_2_ batteries.

Redox mediators (RMs) represent a promising solution to this problem, with numerous studies demonstrating their ability to reduce charging overpotentials and extend cell lifetime.^20–33^ The RM acts as an electrolyte-soluble catalyst, promoting the intended Li_2_O_2_ formation on discharge and/or its oxidation on charge. A large body of work has focussed on charge RMs for Li–O_2_ cells, wherein the mechanism for mediated Li_2_O_2_ oxidation involves electrochemical oxidation of the RM (to RM^+^) at the positive electrode, followed by RM^+^-driven coupled chemical oxidation of solid Li_2_O_2_, evolving O_2_ gas and Li^+^. Therefore, the cell charge potential depends largely on the oxidation potential of the RM, thus enabling a decrease in charge overpotentials. Considering only charge mediators, the chemical space that has been explored for candidate RMs is highly diverse, ranging from organic molecules^20,22–26^ to organometallic^27–29^ and halide-based compounds.^30–33^ Organic RMs are particularly promising due to synthetic versatility, allowing for careful control of the RM redox potential, and indeed, some of the most promising RMs reported are in this category.

Among the explored organic RMs, the majority are cyclic and contain at least one nitrogen atom.^34^ The nitroxide-based family of RMs are well-known in the research community, with most having been evaluated in terms of thermodynamics (RM redox potentials) and kinetics (for reaction between RM^+^ and Li_2_O_2_).^35,36^ 2,2,6,6-Tetramethylpiperidinyloxyl (TEMPO) is a widely studied RM in this sub-class, with Bergner *et al.* being the first to report its use as a charge RM in Li–O_2_ cells operating with lithium bis(trifluoromethanesulfonyl)imide (Li[TFSI]) in diglyme as the base electrolyte.^20^ In this system, the redox potential for the TEMPO^+^/TEMPO couple (*E*_1/2_(TEMPO^+^/TEMPO)) is *ca.* 3.75 V *vs.* Li^+^/Li. Subsequently, TEMPO has primarily been studied as a charge RM in ether-based electrolytes. There are little to no reports of TEMPO in other electrolytes, such as those based on dimethylsulfoxide (DMSO), sulfolane and ionic liquids (ILs) (*e.g.*, pyrrolidinium-based ILs), despite unmediated electrolytes based on each of these solvents having been explored for Li–O_2_ cells.^37–41^ For unexplored electrolyte solvent systems, it is crucial to verify that the selected RM is driving Li_2_O_2_ oxidation, which cannot be confirmed solely through galvanostatic cycling profiles. Instead, an online approach is required, such as differential electrochemical mass spectrometry (DEMS)-based approaches and *operando* internal cell pressure measurement. DEMS analysis of Li–O_2_ cell chemistry has been widely reported for a range of electrolytes,^17,18,41^ and *operando* pressure monitoring has also been employed to study Li–O_2_, Na–O_2_ and Li-ion battery chemistries.^42–46^ Although the former provides access to direct chemical information (*i.e.*, identifying the types of gases evolved during charging), which cannot be discerned from *operando* pressure measurements, the advantage of online pressure monitoring is that gas consumption/evolution can be tracked for the duration of the cell's lifetime. While DEMS has been applied to study gas evolution during cycling of Li-ion cells over many hours (>100 h),^47^ in the context of DEMS studies focussing on Li–O_2_ cell chemistry, such measurements are generally more focussed on a single discharge–charge cycle or even a half-cycle.^20,26^ Therefore, in this work, emphasis is placed on the importance of incorporating *operando* pressure measurements for studying mediated Li–O_2_ cells in combination with *ex situ* characterisation techniques, such as chemical titrations for Li_2_O_2_ yield determination, for the exploration of novel electrolyte formulations.

In this work, the viability of TEMPO as a charge RM is explored in a series of electrolytes based on sulfolane, DMSO, 1-methyl-1-propylpyrrolidinium [TFSI]^−^ ([Pyrr_13_][TFSI]) and diglyme. In-depth studies involving (i) chemical titrations for Li_2_O_2_ yield determination, (ii) current density and internal cell pressure variations, and (iii) *operando* pressure monitoring were conducted on Li–O_2_ cells with a TEMPO-containing sulfolane-based electrolyte to assess the efficacy of TEMPO as a charge RM in this system, with comparison to a diglyme-based electrolyte. Several different analyses were performed on the *operando* pressure data to extract gas consumption/evolution rates and the ratio of moles of charge passed to moles of gas consumed/evolved over a discharge/charge half-cycle, serving as useful markers of cycle-to-cycle evolution of parasitic chemistry. Lastly, an approach combining differential capacity analyses with internal cell pressure measurements as a function of cell potential served as a versatile indicator of RM activity loss during cycling in sulfolane- and diglyme-based electrolytes. These results demonstrate how *operando* pressure data can be a useful tool to screen novel RM-containing electrolyte formulations.

## Experimental

### Materials

Lithium bis(trifluoromethane)sulfonylimide (Li[TFSI], 99%+), 1-propyl-1-methylpyrrolidinium [TFSI] ([Pyrr_13_][TFSI], 99.9%) and 1-butyl-1-methylpyrrolidinium [TFSI] ([Pyrr_14_][TFSI], 99.9%) were purchased from Solvionic, France. Sulfolane (99%) was purchased from Alfa Aesar, US and dimethylsulfoxide (DMSO, anhydrous, 99.9%) from ROMIL, UK. Diglyme (anhydrous, 99.5%), 1.9–2.1% titanium(iv) oxysulfate solution (prepared according to DIN 38 409, part 15, DEV-18), polytetrafluoroethylene (PTFE, 60 wt% dispersion in H_2_O) and silver trifluoromethanesulfonate (Ag[OTf], ≥99%) were purchased from Merck, UK. 2,2,6,6-Tetramethylpiperidinyloxyl (TEMPO, 99%) was purchased from Fluorochem, UK. Ketjenblack EC-600JD was purchased from MSE Supplies, US. Isopropanol (99%) and lithium peroxide (95%) was purchased from Fisher, UK. Glass fibre (Whatman Grade GF/F) was purchased from Lab-Shop, UK. Alumina powder (Buehler, US) of varying particle sizes (1.0, 0.3 and 0.05 μm) was used to polish the glassy carbon working electrode for cyclic voltammetry measurements. All molecular solvents were successively dried over freshly activated 3 Å molecular sieves. Li[TFSI] was dried at 10^−2^ mbar at 120 °C for 24 h, followed by further drying at 10^−5^ mbar for 48–72 h at the same temperature. Glass fibre separators were washed with ethanol several times, dried under vacuum at 110 °C for 15 h in a tube oven (Buchi, Switzerland) and then transferred to an Ar-filled glovebox without exposure to air. All other materials were used as received.

### Electrolyte preparation

All electrolytes were prepared and stored in an Ar-filled glovebox (H_2_O and O_2_ levels below 0.1 ppm). Electrolytes for cyclic voltammetry experiments were prepared volumetrically, containing 10 mM TEMPO and 1 M Li[TFSI] dissolved in diglyme, sulfolane, DMSO or [Pyrr_13_][TFSI]. For Li–O_2_ cell studies, electrolytes based on sulfolane and diglyme solvents with Li[TFSI] and with or without TEMPO were selected. A minimum amount of each electrolyte (∼0.7 mL) was prepared and stored in an Ar-filled glovebox and used within 2 months in order to minimise H_2_O accumulation. The water content of all electrolytes was confirmed to be <15 ppm by coulometric Karl Fischer titration performed in an Ar-filled glovebox. The mole fraction (*x*) ratio of solvent (*x*_solvent_) to Li[TFSI] (*x*_Li[TFSI]_) was fixed at *x*_solvent_ : *x*_Li[TFSI]_ = 9 : 1 and the TEMPO dosing was 25 mmol_TEMPO_ kg_solvent_^−1^. This molal concentration equates to *ca.* 20–25 mmol dm^−3^ TEMPO. Estimation of the TEMPO concentration in terms of molarity is made to allow for comparison to literature and is based on considering solely the volume contribution from the solvent and that of the solvent and Li[TFSI] salt. Table 1 summarises the electrolyte formulations for Li–O_2_ cell studies.

**Table 1:** Electrolyte formulations explored in Li–O_2_ cells^a^

Entry	Electrolyte components	*x*_solvent_ : *x*_Li salt_	Molality		
			mol_solutes_ kg_solvent_^−1^	mol_Li salt_ kg_solvent_^−1^	mol_additives_ kg_solvent_^−1^
1	Li[TFSI]	9 : 1	0.828	0.828	—
	Diglyme				
2	TEMPO	9 : 1	0.853	0.828	0.0249
	Li[TFSI]				
	Diglyme				
3	Li[TFSI]	9 : 1	0.925	0.925	—
	Sulfolane				
4	TEMPO	9 : 1	0.945	0.925	0.0204
	Li[TFSI]				
	Sulfolane				

a *x* denotes the mole fraction.

### Cyclic voltammetry

Cyclic voltammetry was performed using a glassy carbon (GC) working electrode (WE), a Pt counter electrode (CE) and either a Li metal or silver triflate/silver (Ag[OTf]/Ag) reference electrode (RE). The Ag[OTf]/Ag RE, based on a design by Snook *et al.*,^48^ comprised a silver wire immersed in a glass capillary filled with 100 mM Ag[OTf] in [Pyrr_14_][TFSI]. The RE electrolyte was isolated from the analyte electrolyte by a porous glass frit. The GC WE was cleaned and polished after every experiment on microfibre cloth (Buehler, US) wetted with slurries of alumina (particle sizes: 1.0, 0.3 and 0.05 μm) in ultrapure water. Cyclic voltammetry was performed on a SP-300 potentiostat (Bio-Logic, France).

### Carbon black electrode fabrication

Electrodes for Li–O_2_ cells were prepared by spray coating a slurry of carbon black (Ketjenblack EC-600JD) and PTFE (80 : 20 wt%) in isopropanol on to glass fibre under gentle heating at 30 °C. The slurry was stirred vigorously for 24 h prior to spray coating on to glass fibre. After spray coating, *Ø* 10 mm electrodes were punched out, dried at 110 °C for 15 h in a tube oven (Buchi, Switzerland) and then transferred to an Ar-filled glovebox without exposure to air. The carbon black loading was 1.3 ± 0.1 mg_c_ cm^−2^ (1.0 ± 0.1 mg_c_).

### Lithium–oxygen cell assembly and cycling

Standard Li–O_2_ cells were assembled in an Ar-filled glovebox (O_2_, H_2_O <0.1 ppm) and contained the following stack: stainless steel mesh current collector, carbon black positive electrode (prepared as described above), glass fibre separator (*Ø* 12.7 mm) soaked with electrolyte and polished Li metal (*Ø* 12 mm) negative electrode. Standard Li–O_2_ cells contained a polyether ether ketone (PEEK)-lined cell body (Microplas Mouldings Ltd. UK) that housed the electrode stack, with the positive electrode side of the cell exposed to an O_2_ reservoir contained within stainless steel tubing. For the standard cell, quarter-turn gas inlet/outlet valves enabled purging of the cell with pure O_2_ before cycling (see Fig. S1† for images of the standard Li–O_2_ cells used in this work). After assembly, the cell was taken out of the glovebox and purged with high purity O_2_ gas (N5.5, BOC, UK) at 1.25 bar_A_ (absolute pressure) before being sealed at 1.3 bar_A_. The cycling protocol was initiated following a rest period of 8 h. The pressure cell, containing the same electrode stack within an identical PEEK-lined cell body, was assembled in the same way, except that cells were sealed at 1.5 bar_A_ and rested for 18–24 h to allow for a stable leak rate (*ca.* 10^−5^ bar h^−1^) to be established before cycling. Cells were cycled at a current density of 80 mA g_c_^−1^ (0.11 ± 0.01 mA cm^−2^) with discharge and charge potential cut-offs of 2.0 and 4.5 V *vs.* Li^+^/Li, respectively, and a capacity-limit of either 500 (0.7 ± 0.1 mAh cm^−2^) or 1000 mAh g_c_^−1^ (1.3 ± 0.1 mAh cm^−2^). For the internal cell pressure and current density variations, cells were cycled at 1.3, 1.75 and 2.2 bar_A_ (absolute pressure), and 80, 160 (0.21 ± 0.02 mA cm^−2^) and 320 mA g_c_^−1^ (0.42 ± 0.03 mA cm^−2^), respectively. Standard Li–O_2_ cells were cycled on a BCS-805 battery cycler (Bio-Logic, France) and the pressure cell on a SP-150 potentiostat (Bio-Logic, France).

### UV-vis titration for Li_2_O_2_ yield determination

Li–O_2_ cells with carbon black electrodes (prepared as described above) were discharged to 1000 mAh g_c_^−1^ at 80 mA g_c_^−1^. Cells were then purged with Ar before being taken into an Ar-filled glovebox for disassembly and electrode extraction. The carbon electrodes were dried under vacuum in the glovebox antechamber for 2 h and then placed into septum-capped vials. The electrodes were then taken out of the glovebox and treated with a 1 : 1 solution of TiOSO_4_ : H_2_O, producing a yellow solution due to formation of a [TiO_2_]^2+^ complex. After reacting for 30 min, an aliquot of the solution was transferred to a cuvette for determination of the absorbance at *λ*_max_ = 407 nm by UV-Vis spectroscopy (Evolution 201 UV-Vis Spectrophotometer, Thermo Fisher Scientific, US). The Li_2_O_2_ yield was determined using a calibration curve (Fig. S2†) prepared with known amounts of commercial Li_2_O_2_, taking into account a quoted purity of 95%. For each electrolyte, yield tests were performed in duplicate.

## Results and discussion

### Electrolyte-dependent redox behaviour of TEMPO

**Fig. 1:**
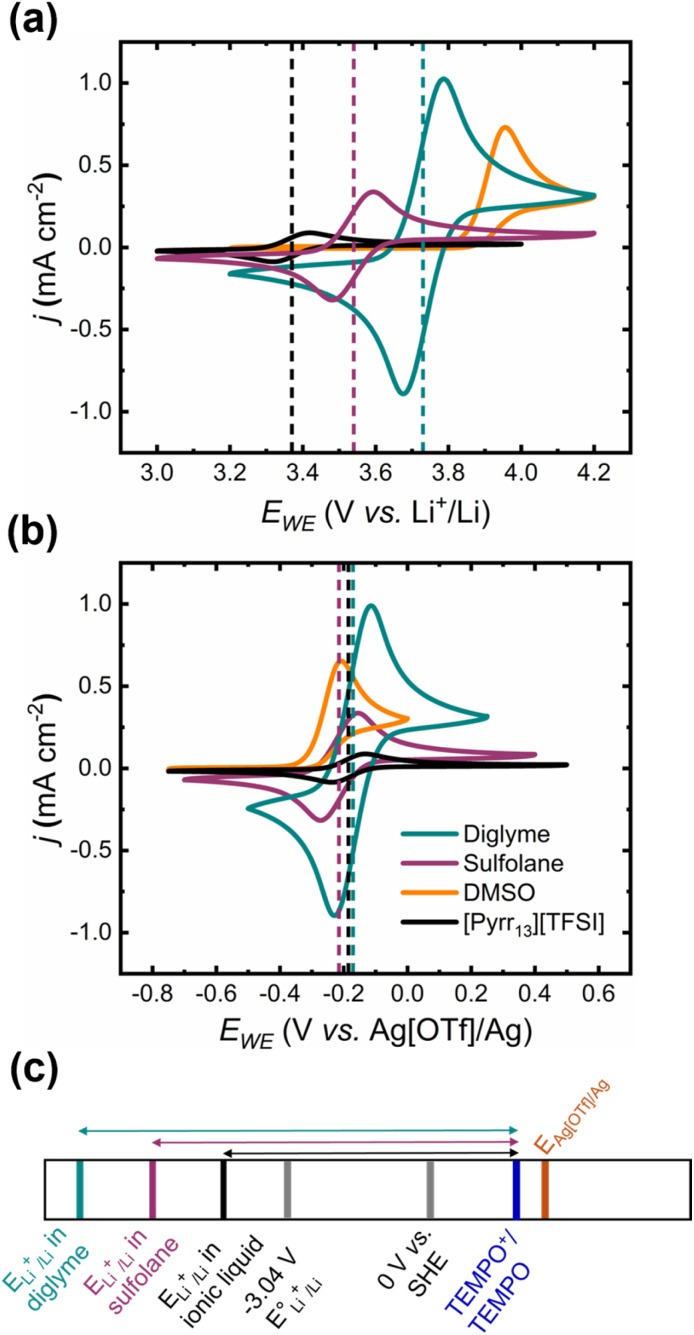
Cyclic voltammograms (CVs) of 10 mM TEMPO in various electrolytes based on diglyme (cyan), sulfolane (purple), DMSO (orange) and [Pyrr_13_][TFSI] (black), with 1 M Li[TFSI] as the supporting electrolyte in all systems. CVs were acquired using a glassy carbon working electrode (GC WE), Pt counter electrode (CE), and either a (a) lithium metal or (b) silver triflate/silver (Ag[OTf]/Ag) reference electrode (RE) at a scan rate of 50 mV s^−1^ under an Ar atmosphere. The potential scale below (c) highlights relative differences in the Li reduction potential as a function of the electrolyte and how this impacts the measured *E*_1/2_(TEMPO^+^/TEMPO) values (magnitude of double-headed arrows) *vs.* Li^+^/Li.

The redox behaviour of TEMPO in several molecular solvent- and ionic liquid-based electrolytes under an inert atmosphere was first explored by cyclic voltammetry. Fig. 1 shows the redox behaviour of TEMPO in a range of electrolytes with Li[TFSI] as the supporting electrolyte under an Ar atmosphere at a glassy carbon working electrode (GC WE). The CVs were measured with a Pt and Li metal counter electrode (CE) and reference electrode (RE), respectively. Significant differences in the TEMPO redox potential (*E*_1/2_(TEMPO^+^/TEMPO)), with a range of 360 mV, are observed in the CVs measured with a Li metal RE. These shifts are attributed primarily to the differences in Li reduction potential as a function of the electrolyte, rather than a change in *E*_1/2_(TEMPO^+^/TEMPO), as depicted by the potential scale shown in Fig. 1c. This is demonstrated in CVs performed using a silver triflate/silver (Ag[OTf]/Ag) RE (see Experimental for details of RE assembly). This RE is separated from the analyte electrolyte by a porous glass frit, maintaining a more electrolyte-independent reference potential. As such, *E*_1/2_(TEMPO^+^/TEMPO) varies by <50 mV between the different solvent systems. However, the changes in *E*_1/2_(TEMPO^+^/TEMPO) observed with the Li metal RE are still of practical significance for Li–O_2_ cells where Li metal serves as the RE and CE.

As has been reported previously, the TEMPO^+^/TEMPO redox couple is reversible in the diglyme-based electrolyte.^20^ However, upon oxidation in the dimethylsulfoxide (DMSO)-based electrolyte, the corresponding reduction peak is absent, suggesting that TEMPO^+^ is irreversibly consumed *via* a coupled chemical reaction with DMSO. If the scan rate is increased (Fig. S3a†), partial recovery of the TEMPO^+^ to TEMPO reduction peak can be observed, indicative of a finite reaction rate for TEMPO^+^ consumption with DMSO. Furthermore, when the Li[TFSI] concentration is increased such that the ratio of Li^+^-coordinated DMSO (less reactive, contact ion pairs) to free DMSO (solvent-separated ion pairs) increases,^49^ a clear TEMPO^+^ reduction peak is observed on the reverse sweep (Fig. S3b†), but the asymmetry of the peak currents confirm TEMPO^+^ is still being chemically consumed. These results confirm that TEMPO^+^ is unstable in DMSO-based electrolytes, undergoing decomposition reactions with the DMSO solvent. Conversely, the TEMPO^+^/TEMPO couple is chemically reversible in the sulfolane-based electrolyte, where the sulfur atom is in its highest oxidation state and so cannot be oxidised further, as well as in a pyrrolidinium-based ionic liquid ([Pyrr_13_][TFSI]) electrolyte.

The redox potential of TEMPO^+^/TEMPO *versus* Li^+^/Li, *E*_1/2_(TEMPO^+^/TEMPO), increases in the following order in terms of the electrolyte solvent: [Pyrr_13_][TFSI] < sulfolane < diglyme (Table 2). Assuming that changes in *E*_1/2_(TEMPO^+^/TEMPO) are due to the Li|electrolyte interface changing, this suggests that Li^+^ stabilisation by solvent coordination is greatest in the diglyme-based electrolyte and lowest in [Pyrr_13_][TFSI]-based system. From a purely thermodynamic perspective, a lower *E*_1/2_(TEMPO^+^/TEMPO) value that is still above the theoretical formation/decomposition potential for Li_2_O_2_ (2.96 V *vs.* Li^+^/Li) is favourable for low overpotential RM-mediated charging of Li–O_2_ cells, making the ionic liquid (IL)-based electrolyte the ideal candidate system. However, the high viscosity of the neat ionic liquid (58.7 mPa s at 25 °C),^50^ exacerbated further still by dissolution of Li salts, would likely result in large kinetic overpotentials in Li–O_2_ cells with this electrolyte. Therefore, the efficacy of TEMPO as a charge RM was assessed in a sulfolane-based electrolyte in Li–O_2_ cells, with comparison to the TEMPO-containing diglyme-based system.

**Table 2:** Summary of *E*_1/2_(TEMPO^+^/TEMPO) in different electrolytes as determined by cyclic voltammetry using a GC WE, Pt CE and either a lithium metal or Ag[OTf]/Ag RE at a scan rate of 50 mV s^−1^ under an Ar atmosphere

Base electrolyte with 10 mM TEMPO	*E*_1/2_(TEMPO^+^/TEMPO) *vs.* Li^+^/Li (V)	*E*_1/2_(TEMPO^+^/TEMPO) *vs.* Ag[OTf]/Ag (V)
1 M Li[TFSI] in diglyme	3.73	−0.17
1 M Li[TFSI] in sulfolane	3.53	−0.22
1 M Li[TFSI] in [Pyrr_13_][TFSI]	3.37	−0.19

### First galvanostatic discharge–charge cycle and lithium peroxide yield quantification

**Fig. 2:**
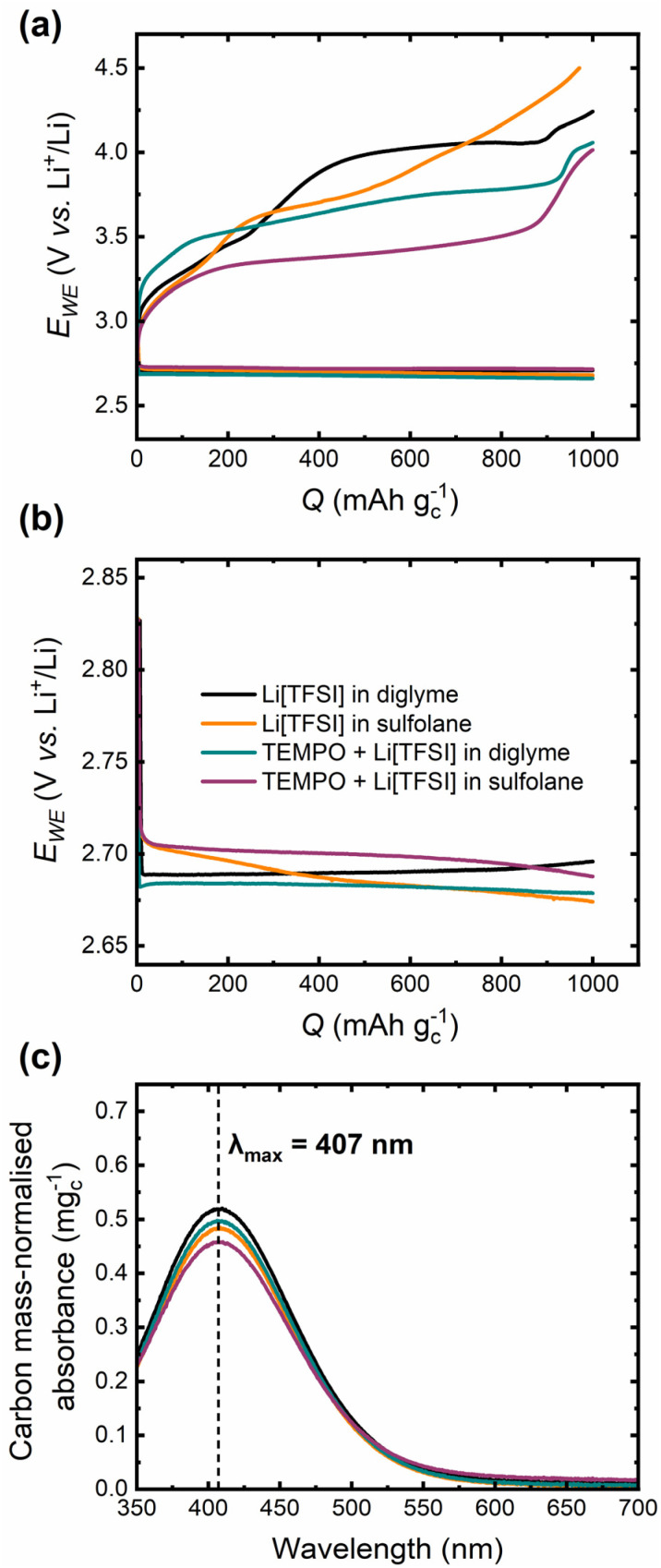
(a) First capacity-limited (1000 mAh g_c_^−1^) cycle in standard Li–O_2_ cells in mediated (25 mmol_TEMPO_ kg_solvent_^−1^) and unmediated diglyme- and sulfolane-based electrolytes at 80 mA g_c_^−1^, where the mole fraction (*x*) ratio of solvent: Li[TFSI] salt was *x*_solvent_ : *x*_Li[TFSI]_ = 9 : 1 (see Experimental section). (b) Single 1000 mAh g^−1^ discharge in the four different electrolytes at 80 mA g_c_^−1^. Electrodes from these cells were subjected to the chemical titration for Li_2_O_2_ yield quantification. (c) The associated UV-Vis spectra of aliquots of aqueous solutions containing the discharged electrodes treated with a 1 : 1 TiOSO_4_ (in H_2_SO_4(aq)_) : H_2_O solution for determination of Li_2_O_2_ yield. The absorbance is normalised by the carbon black mass on the positive electrode.

The first cycle in Li–O_2_ cells under a galvanostatic, capacity-limited cycling regime in mediated and unmediated diglyme- and sulfolane-based electrolytes with carbon black-based positive electrodes (*versus* Li metal) is shown in Fig. 2a. The “standard” Li–O_2_ cell used for these experiments consisted of stainless-steel Swagelok components, with an insulating sheath within the cell body housing the electrode stack. The cell was connected to a high purity O_2_ gas line to enable purging with O_2_ before cycling (see Experimental for a more detailed description of the cell and Fig. S1† for images of the standard cell configuration). Cell discharge proceeds with minimal potential variation across the four electrolytes, with plateaus at *ca.* 2.70–2.75 V *vs.* Li^+^/Li. However, there are significant differences in the charge profiles for the different electrolyte systems. Incorporating TEMPO into both electrolytes results in a significant decrease in the charge overpotential (*η*_ch_); at 80% of the charge capacity limit, the unmediated electrolytes exhibit overpotentials >1 V, but introducing the TEMPO RM reduces observed *η*_ch_ to *ca.* 0.8 V and 0.5 V for diglyme and sulfolane-based electrolytes, respectively. However, in both mediated systems there is a sharp rise in charge potential beyond this point, which could be related to the decreasing ability of TEMPO^+^ to oxidise a continually decreasing amount of Li_2_O_2_ left on the carbon electrode.^20^ The lower charge overpotential in the mediated sulfolane electrolyte compared to the corresponding diglyme system is consistent with the TEMPO^+^/TEMPO redox potentials determined from the CVs with a Li metal RE (Fig. 1a), demonstrating that these measured redox potentials are practically relevant for mediated Li–O_2_ cells.

To confirm that Li_2_O_2_ formed on the carbon black electrodes used, cells were discharged to 1000 mAh g^−1^ (Fig. 2b) and the extracted electrodes treated with a 1 : 1 TiOSO_4_ (in H_2_SO_4_/H_2_O) : H_2_O solution. Water reacts with Li_2_O_2_ to form lithium hydroxide (LiOH) and hydrogen peroxide (H_2_O_2_). Subsequently, the Li_2_O_2_ yield was determined by UV-Vis spectroscopy of the resulting aliquots (Fig. 2c), quantified using a calibration curve derived from standard solutions of Li_2_O_2_ in water (Fig. S2†). Across the four electrolytes, yields ranged between 70–80%, with the diglyme systems forming more Li_2_O_2_ than the sulfolane-based electrolytes (Table 3). The yields for the diglyme-based electrolytes are consistent with previous reports for ether-based systems.^51,52^ However, no prior reports on the application of this chemical titration method for sulfolane-based electrolytes could be found. Therefore, the reported yields in this work for the sulfolane-based systems are discussed in the context of *operando* internal cell pressure measurements later.

**Table 3:** Summary of Li_2_O_2_ yields in mediated and unmediated diglyme- and sulfolane-based electrolytes after discharging to 1000 mAh g_c_^−1^ at 80 mA g_c_^−1^

Electrolyte	% Yield
Li[TFSI] in diglyme	80.3 ± 0.4
Li[TFSI] in sulfolane	73.8 ± 3.2
TEMPO + Li[TFSI] in diglyme	80.3 ± 3.7
TEMPO + Li[TFSI] in sulfolane	71.8 ± 1.1

### Current density and internal cell pressure variations

Having confirmed that Li_2_O_2_ is the primary discharge product, the effects of internal cell pressure and current density on discharge/charge overpotentials, discharge capacity, and cell rechargeability was evaluated. These two parameters were chosen as the former has been shown to influence discharge product morphology, and therefore, discharge/charge overpotentials,^9^ while the latter impacts oxygen diffusivity and concentration in the electrolyte.^53^ For these parameter studies, cells were limited to 10 discharge/charge cycles under a galvanostatic, capacity-limited (1000 mAh g_c_^−1^) regime. The baseline current density and absolute internal cell pressure was 80 mA g_c_^−1^ (0.11 ± 0.01 mA cm^−2^) and 1.3 bar_A_, respectively. The current density was increased to 160 mA g_c_^−1^ (0.21 ± 0.02 mA cm^−2^) and 320 mA g_c_^−1^ (0.42 ± 0.03 mA cm^−2^), and the cell pressure was varied to 1.75 and 2.2 bar_A_.

**Fig. 3:**
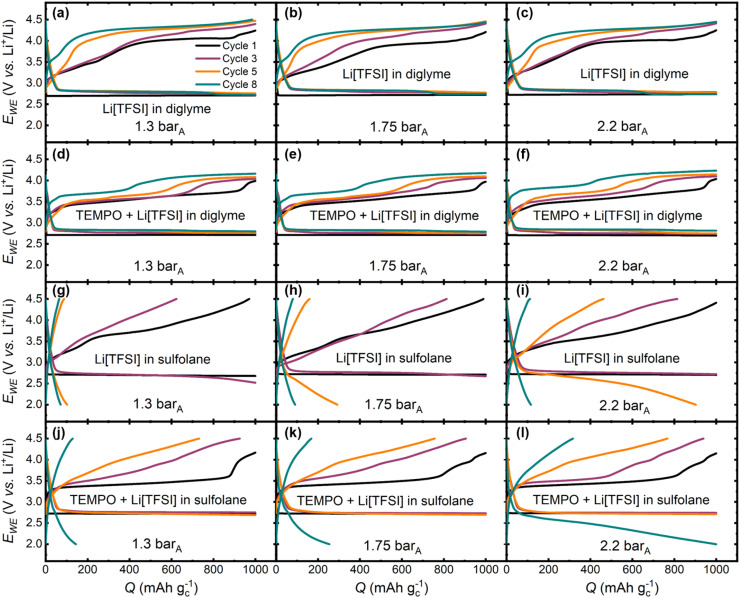
Galvanostatic (80 mA g_c_^−1^), capacity-limited (1000 mAh g^−1^) cycles 1 (black), 3 (purple), 5 (orange) and 8 (cyan) of Li–O_2_ cells sealed at internal O_2_ gas pressures of 1.3, 1.75 and 2.2 bar_A_ for (a–c) Li[TFSI]-diglyme, (d–f) TEMPO-Li[TFSI]-diglyme, (g–i) Li[TFSI]-sulfolane and (j–l) TEMPO-Li[TFSI]-sulfolane electrolytes. See Fig. S4–7† for all cycles across the four electrolytes. [TEMPO] = 25 mmol_TEMPO_ kg_solvent_^−1^, *x*_solvent_ : *x*_Li[TFSI]_ = 9 : 1, where *x* = mole fraction.

Fig. 3 shows the effect of pressure variation across the four electrolytes studied in this work at cycles 1, 3, 5 and 8. Considering the unmediated diglyme electrolyte, the pressure variations have no significant effect on discharge/charge overpotentials (Fig. 3a–c) without TEMPO. The same is true for the TEMPO-mediated system (Fig. 3d–f), but at 2.2 bar_A_ there is an increased overpotential on charge associated with electrochemical TEMPO oxidation, which is at its greatest in cycle 10 (*ca.* 150 mV higher relative to cells cycled at 1.3 and 1.75 bar_A_, Fig. S4†). This could be related to an increased driving force required for mediated Li_2_O_2_ oxidation into an atmosphere with a high O_2_ gas concentration compared to the lower pressure measurements.

Significant differences in the discharge/charge profiles are observed as a function of pressure with the sulfolane-based electrolytes (Fig. 3g–l). In both the mediated and unmediated systems, achievable discharge capacities and rechargeability are improved with increasing pressure, which could be related to an increased dissolved O_2_ concentration. Furthermore, relative to the unmediated sulfolane electrolyte, rechargeability and discharge capacities are improved at all pressures with TEMPO present. Although beyond the scope of the parameter study, it is also worth noting that differences in the discharge/charge profiles across the diglyme electrolytes may occur beyond 10 cycles, whereas the capacity-fade and polarisation on charge is more severe in the sulfolane-based electrolytes, such that the influence on cyclability can be observed within 10 cycles.

**Fig. 4:**
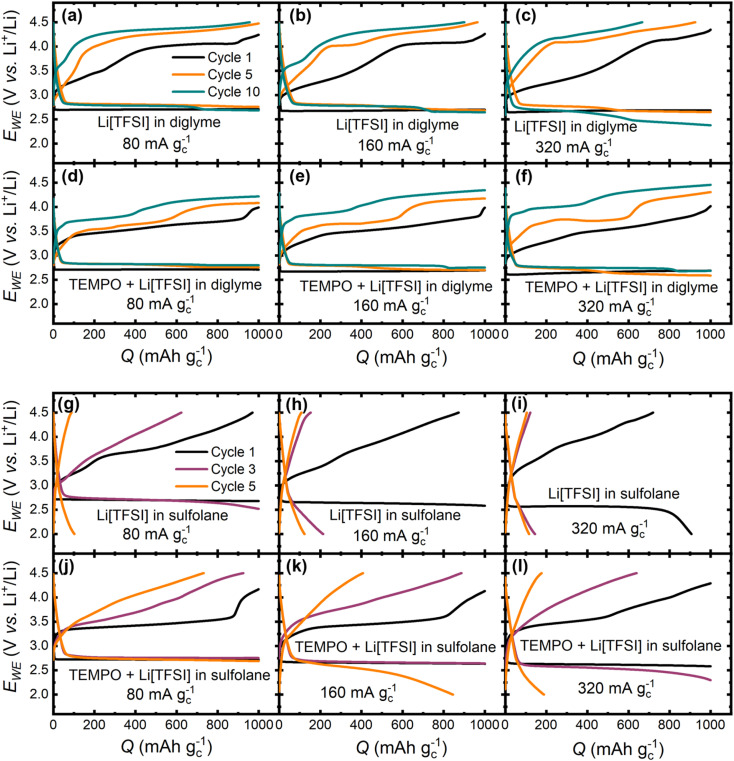
Galvanostatic, capacity-limited (1000 mAh g^−1^) cycles of Li–O_2_ cells at varying current densities: 80, 160 and 320 mA g_c_^−1^. For (a–c) Li[TFSI]-diglyme and (d–f) TEMPO-Li[TFSI]-diglyme electrolytes cycles 1 (black), 5 (orange) and 10 (cyan) are shown, and, due to the lower cyclability of sulfolane electrolytes relative to diglyme systems, cycles 1 (black), 3 (purple) and 5 (orange) are presented for (g–i) Li[TFSI]-sulfolane and (j–l) TEMPO-Li[TFSI]-sulfolane electrolytes. All cells were sealed at 1.3 bar_A_. See Fig. S8–11† for all cycles across the four electrolytes. [TEMPO] = 25 mmol_TEMPO_ kg_solvent_^−1^, *x*_solvent_ : *x*_Li[TFSI]_ = 9 : 1, where *x* = mole fraction.

Following the pressure variations, the effect of applied current density on cell cycling was explored. Fig. 4 shows the galvanostatic cycling profiles for Li–O_2_ cells with unmediated and mediated diglyme- and sulfolane-based electrolytes. For the diglyme systems, discharge overpotentials (*η*_dch_) increase with increasing current density. However, in cycle 1 for the unmediated diglyme electrolyte (Fig. 4a), the onset potential for Li_2_O_2_ oxidation on charge decreases (decreased *η*_ch_) with increasing current density. This trend is consistent with previous reports and is likely a consequence of the variation in Li_2_O_2_ morphology formed on discharge as a function of current density. For example, Adams *et al.*^9^ demonstrated that in a Li[TFSI]-tetraethylene glycol dimethyl ether electrolyte, nanocrystalline toroidal Li_2_O_2_ aggregates form at low discharge rates, whereas higher current densities yield thin film-like Li_2_O_2_, with oxidation of the latter being more facile due to improved electrical contact with the positive electrode. However, this trend does not persist in later cycles, likely due to variations in Li_2_O_2_ oxidation efficiency and the accumulation of parasitic products.

The same first cycle trend is observed with the TEMPO-mediated diglyme electrolyte, with the decreasing charge onset potentials reflecting the ease with which direct electrochemical oxidation of Li_2_O_2_ occurs. This is not surprising as these onset potentials are below the oxidation plateau ascribed to electrochemical TEMPO oxidation. Thus, mediated Li_2_O_2_ oxidation is not expected to be the dominant process at this stage since TEMPO is not expected to affect the discharge process significantly. In cycles 5 and 10, charge overpotentials increase with increasing current density. For the potential plateau at *ca.* 3.6–3.8 V *vs.* Li^+^/Li (depending on cycle number and current density), ascribed to electrochemical TEMPO oxidation to TEMPO^+^, the increase in overpotentials with current density could be due to cell potential being under mass transport control, *i.e.*, diffusion of TEMPO to the carbon electrode, and the accumulation of parasitic products at this electrode.^23,24^ The capacity contribution of the TEMPO oxidation plateau decreases on cycling with a higher potential plateau (at *ca.* 3.8–4.3 V *vs.* Li^+^/Li depending on current density) making up the difference. As has been reported previously, the growth of this higher potential plateau is related to increasing decomposition of parasitic products,^20^ which eventually contributes to *ca.* 60% of the total charge capacity by cycle 10, suggesting accumulation of parasitic products on cycling.

In the sulfolane-based electrolytes (Fig. 4g–l), discharge and charge overpotentials increase with increasing current density across all cycles with and without TEMPO. This is likely due to the higher viscosity of sulfolane (10.6 mPa s at 30 °C)^54^ compared to diglyme (1.1 mPa s 20 °C),^55^ and therefore, mass transport of all species required for Li_2_O_2_ formation/decomposition govern cell potential from the first cycle. It is also important to note that incorporation of TEMPO improves achievable discharge capacities and rechargeability across all current densities, relative to the unmediated electrolyte. At 80 and 160 mA g_c_^−1^, in the TEMPO-mediated system, the sharp rise in potential towards the end of charge in cycle 1 likely reflects the challenge of oxidising small amounts of Li_2_O_2_, as was observed in the mediated diglyme electrolyte. However, at 320 mA g_c_^−1^, the rise in potential is not as steep and initiates at *ca.* 50% of the charge capacity. As this increase in overpotential occurs midway through the charge step, it cannot be explained by a decreasing amount of Li_2_O_2_. Instead, it is likely a combined effect of (i) kinetic overpotentials induced by the diffusion limitations of TEMPO in the more viscous sulfolane-based electrolyte, and (ii) an increasing proportion of direct electrochemical oxidation of Li_2_O_2_ at this higher rate.

Across both diglyme- and sulfolane-based electrolytes, there is also the possibility of the rate of reaction between TEMPO^+^ and Li_2_O_2_ not being fast enough to sustain the applied current density, thus resulting in the observed polarisation on charge. However, Chen and co-workers have demonstrated that mediated Li_2_O_2_ oxidation kinetics can sustain areal current densities on the order of 100 mA cm^−2^, more than 250× greater than the highest equivalent areal current density applied in this work, at charge overpotentials <0.1 V based on a model of a porous electrode filled with Li_2_O_2_.^35^ Importantly, nitroxide-based RMs exhibited the highest apparent reaction rates for Li_2_O_2_ oxidation by RM^+^ across the tested RMs. Therefore, it is concluded that this factor is not a major source of polarisation on charge at higher rates. The differences in electrochemical behaviour between the diglyme- and sulfolane-based electrolytes as a function of current density and O_2_ pressure emphasizes the challenge and importance of optimising cycling parameters for Li–O_2_ cells, as the optimal parameters depend strongly on the physicochemical properties of the electrolyte, which can vary significantly between solvent families.

### *Operando* pressure measurements for assessing RM efficacy

The parameter studies discussed in the previous section provide useful insights into the effects of current density and internal cell pressure variations as a function of electrolyte. However, these galvanostatic profiles do not provide a complete picture of the electrochemical reactions occurring in the cell, particularly when charging TEMPO-mediated cells. Additionally, although the UV-Vis titration approach (Fig. 2b and c) confirms the formation of Li_2_O_2_ in all electrolytes used in this work, this approach is limited by the destructive nature of the electrode treatment and does not consider any Li_2_O_2_ that is lost during electrode extraction and/or consumed after formation through parasitic reactions (*e.g.*, formation of Li_2_CO_3_ by reaction of Li_2_O_2_ with the carbon electrode).^17,18^ Furthermore, the chemical titration approach becomes impractical to study Li_2_O_2_ yield beyond the first discharge, as the incomplete oxidation of Li_2_O_2_ on the subsequent charge means that Li_2_O_2_ yield determination on, for example, the second discharge would include Li_2_O_2_ left on the electrode after the first charge. Therefore, an online approach is required to study the Li–O_2_ cell under *operando* conditions. Here, an *operando* pressure-electrochemical cell (hereafter referred to as “pressure cell”) was built using Swagelok parts to monitor internal pressure within the cell headspace over the entire lifetime of the cell, from which the efficacy of TEMPO as a charge RM can be deduced as a function of the electrolyte. The cell headspace was interfaced with a high accuracy pressure transducer, enabling highly sensitive tracking of small pressure changes (<5 mbar) in response to changes in the electrochemical cycling profile. Importantly, *operando* pressure measurements serve as a complimentary, readily accessible technique in the Li–O_2_ cell diagnostic toolkit, further strengthened by the RM efficacy analysis involving correlation of differential capacity with cell pressure changes as described later. A schematic of the cell (Fig. S11†), details of the cell volume calibration (Fig. S12–14†) and a description of the pressure cell data processing procedure (ESI Note 1 and Fig. S15†) are provided in the ESI.† The electrolyte volume (80 μL) and dimensions of the carbon black-based positive electrode used was identical to the ‘standard’ Swagelok-type Li–O_2_ cells used for cell cycling experiments where pressure was not measured. This was done to ensure flooding factors, defined as the ratio of electrolyte volume to active electrode area of the positive electrode, are comparable between the standard and pressure cells (*ca.* 6.3 ± 0.6 nL cm^−2^, calculated using the BET specific surface area of the carbon black material used in this work (Ketjenblack EC-600JD, 1270 m^2^ g^−1^) and a carbon black loading of 1.0 ± 0.1 mg). Unlike with other *operando* methods, where often entirely different cell designs are required (*e.g.*, DEMS), this pressure cell enables Li–O_2_ chemistry to be studied under conditions directly comparable to the standard cell.

**Fig. 5:**
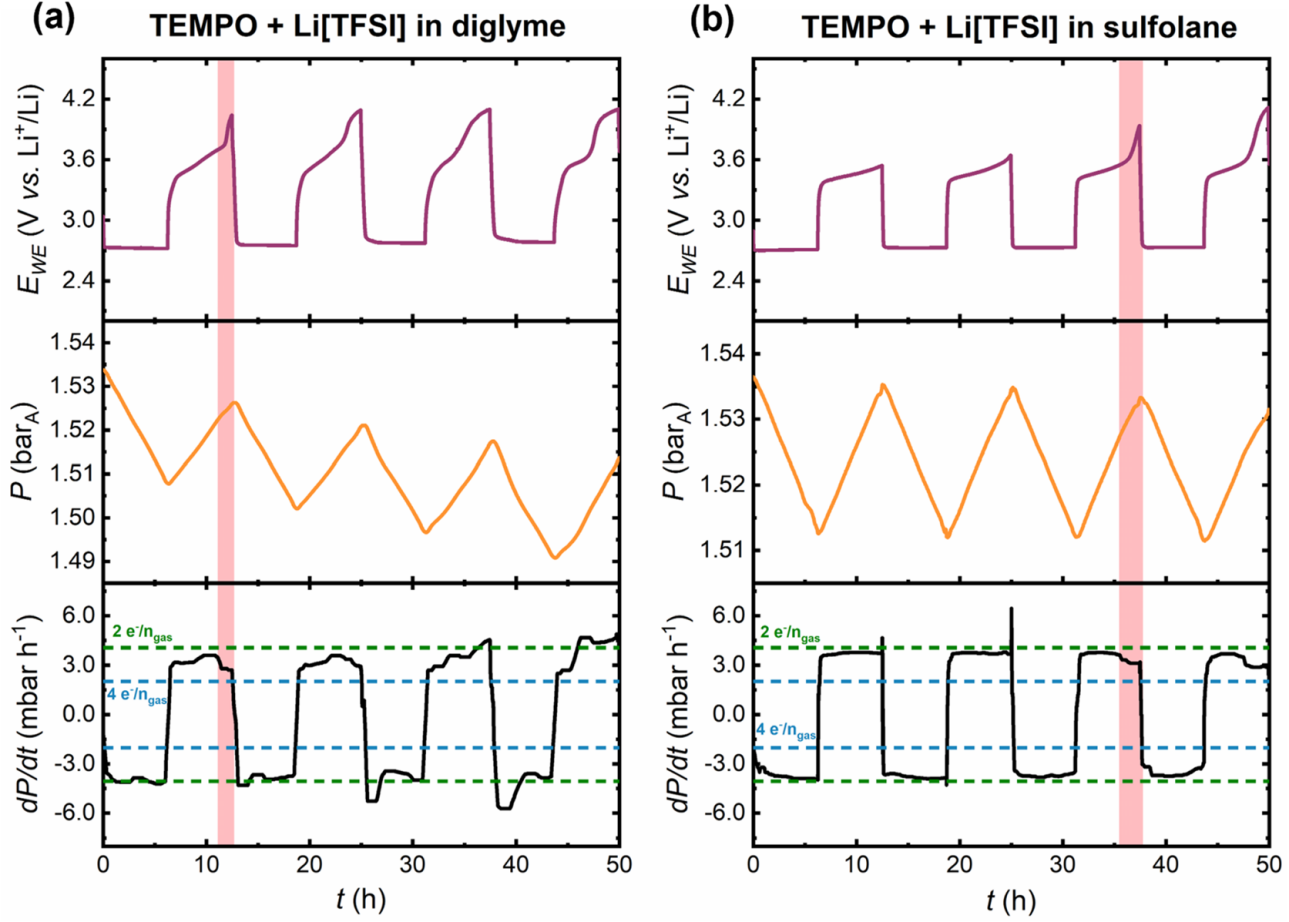
Cycles 1–4 in the pressure cells cycled under a capacity-limited (500 mAh g^−1^) regime at 80 mA g^−1^ in TEMPO-mediated (a) diglyme-and (b) sulfolane-based electrolytes. The RM loading was 25 mmol_TEMPO_ kg_solvent_^−1^ and the mole fraction (*x*) ratio of solvent : Li[TFSI] salt was *x*_solvent_ : *x*_Li[TFSI]_ = 9 : 1. The cell potential (purple), pressure response (orange) and first derivative of the pressure response (black) are shown as a function of time. The dotted green and blue lines indicate the theoretical gas consumption/evolution rates for 2e^−^/*n*_gas_ and 4e^−^/*n*_gas_ processes, respectively. The red shaded regions highlight the sensitivity of the pressure measurement, capturing drops in gas evolution rate that coincide with sharp rises in cell potential, which serve as a marker for decreased RM efficacy.

Fig. 5 shows the first four cycles in the pressure cell with the mediated diglyme- and sulfolane-based electrolytes, wherein the cell potential (top panels), headspace pressure variation (middle panels) and first derivative of the pressure response (bottom panels) are shown as a function of time. In both mediated systems, during the discharge plateau at *ca.* 2.7 V *vs.* Li^+^/Li there is a decrease in pressure, consistent with the consumption of O_2_ gas for the ORR. Charging of Li–O_2_ cells relies on a coupled chemical oxidation wherein electrochemical RM oxidation to RM^+^ is followed by RM^+^ oxidation of Li_2_O_2_, regenerating RM and evolving O_2_ gas. Therefore, it is essential to verify whether this coupled chemical oxidation takes place, and if so, how it evolves from cycle to cycle, particularly when studying novel RM-containing electrolyte formulations. Importantly, this information cannot be explicitly determined from the galvanostatic cycling profiles alone. As Fig. 5b shows, the TEMPO oxidation plateau in the first charge half-cycle in the sulfolane system coincides with gas pressure increase, confirming that the coupled-chemical oxidation process (*i.e.*, RM^+^ + Li_2_O_2_ → RM + 2Li^+^ + O_2(gas)_) is occurring. A similar response is observed in the mediated diglyme system, however, the charge overpotential is greater in the first three charge steps.

Using the ideal gas law and the calibrated cell volume, the average electron-to-gas mole ratio (*n*e^−^/*n*_gas_) can be estimated. Here, ‘‘average’’ refers to *n*e^−^/*n*_gas_ values calculated based on the total pressure change for a discharge/charge half-cycle (see ESI Note 2† for example calculations). For the desired two-electron ORR and OER, the ideal *n*e^−^/*n*_gas_ value should correspond to 2 moles of electrons per mole of gas consumed/evolved (2e^−^/*n*_gas_), assuming that all the gas being evolved is O_2_ and that the only 2e^−^/*n*_gas_ processes are 2-electron ORR and OER. For example, in the first discharge half-cycle, the average *n*e^−^/*n*_gas_ is calculated as 2.06 and 2.09 in the mediated diglyme and sulfolane systems, respectively. This is consistent with the overpotentials on discharge being almost identical. However, in the subsequent charge half-cycle, there is greater polarisation in the mediated diglyme cell as compared to the sulfolane electrolyte; the former polarises to *ca.* 4 V *vs.* Li^+^/Li, whereas the latter remains below 3.6 V *vs.* Li^+^/Li. Consequently, the deviation in the average *n*e^−^/*n*_gas_ ratio for the entire charge step from the ideal value of 2e^−^/*n*_gas_ is greater in the mediated diglyme electrolyte (2.55e^−^/*n*_gas_) relative to the mediated sulfolane-based electrolyte (2.14e^−^/*n*_gas_).

During charging of mediated Li–O_2_ cells, sharp rises in potential are observed, which deviates significantly from the charge plateau ascribed to electrochemical RM oxidation. As described earlier, this may be related to the difficulty in mediated oxidation of small amounts of Li_2_O_2_ left on the carbon electrode and oxidation of parasitic products. This effect is exacerbated on cycling, with a decreasing capacity contribution to the total charge capacity stemming from the TEMPO oxidation plateau, as was observed during cycling in standard Li–O_2_ cells (Fig. 3 and 4). Fig. 5a shows a transition point (see red shaded region) wherein a sharp rise in potential in the 1^st^ charge half-cycle in the mediated diglyme electrolyte, results in a decrease in the slope of the pressure response. This can be clearly seen in the first derivative plots (d*P*/d*t*) showing instantaneous gas consumption/evolution rates and the corresponding ideal rates. The ideal rates were calculated as follows (see ESI Note 3† for example calculations): using the cell volume, the pressure change for consumption/evolution of a known amount (moles) of gas was calculated. As a fixed capacity limit and current density was applied, the time required for a single discharge/charge is known, and therefore, the theoretical gas consumption/evolution rate can be determined. The deviation in the instantaneous gas evolution rate from the ideal rate for a 2e^−^/*n*_gas_ process is indicative of the extent of parasitic chemistry. Crucially, tracking instantaneous gas consumption/evolution rates provides information on when during a given charge half cycle RM efficacy is diminished, which cannot be understood solely from average *n*e^−^/*n*_gas_ values. A sharp rise in potential is also observed in the 3rd charge half-cycle in the mediated sulfolane system (Fig. 5b). In both mediated electrolytes, the instantaneous gas evolution rates decrease during these sharp potential increases (Fig. 5, red highlighted regions), indicating that *n*e^−^/*n*_gas_ > 2, demonstrating that the pressure cell can be used to accurately correlate small changes in cycling behaviour, providing insight into transition points in a half-cycle where RM functionality is reduced.

In cycles 1–4 in both electrolytes, discharge proceeds close to the 2e^−^/*n*_gas_ gas consumption rate. However, charging proceeds closer to the 2e^−^/*n*_gas_ gas evolution rate in the mediated sulfolane electrolyte, which is consistent with the lower charge overpotentials measured in this system. Furthermore, comparing the first four cycles in the corresponding unmediated electrolytes in the pressure cell (Fig. S16a†) with the mediated systems reveals some important differences. While instantaneous gas consumption/evolution rates do not differ significantly in the unmediated and mediated diglyme electrolytes, the overpotentials on charge are reduced with TEMPO present. Incorporation of TEMPO into the sulfolane-based electrolytes brings the instantaneous gas evolution rates closer to the ideal rate with at least a 0.3 V decrease in charge overpotentials in the first four cycles (Fig. 5b and S16b†). However, the question remains how the electrochemical behaviour and pressure responses observed in early cycles evolve with continued cell cycling.

**Fig. 6:**
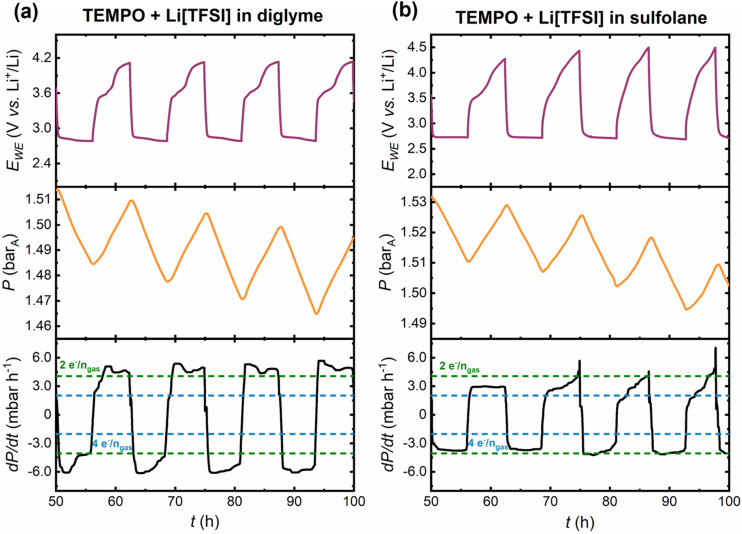
Cycles 5–8 of the pressure cells with TEMPO-mediated (a) diglyme-and (b) sulfolane-based electrolytes. The RM loading was 25 mmol_TEMPO_ kg_solvent_^−1^ and the mole fraction (*x*) ratio of solvent : Li[TFSI] salt was *x*_solvent_ : *x*_Li[TFSI]_ = 9 : 1. The cell potential (purple), pressure response (orange) and first derivative of the pressure response (black) are shown as a function of time. The dotted green and blue lines indicate the theoretical gas consumption/evolution rates for 2e^−^/*n*_gas_ and 4e^−^/*n*_gas_ processes, respectively.

Fig. 6 shows cycles 5–8 in the pressure cell with the mediated electrolytes, where distinct differences in the discharge–charge profiles and associated gas consumption/evolution rates are observed. Firstly, on discharge in the mediated diglyme electrolyte, there is a significant overconsumption of gas, that is, *n*e^−^/*n*_gas_ < 2. This overconsumption of gas in diglyme-based electrolytes has been reported previously by Lepoivre *et al.* suggesting that a discharge reaction involving both O_2_ and CO_2_ consumption was taking place (eqn (1)).^44^

1 4Li^+^ + 2CO_2_(g) + O_2_(g) + 4e^−^ → 2Li_2_CO_3_

Eqn (1) would result in a *n*e^−^/*n*_gas_ ratio of 1.33 and is consistent with the formation of Li_2_CO_3_ due to decomposition reactions associated with the carbon electrode (in contact with Li_2_O_2_) and the electrolyte.^17,18^ Subsequently, at high charging potentials (>4 V *vs.* Li^+^/Li), Li_2_CO_3_ is electrochemically oxidised evolving CO_2_, which is then present to be consumed in the following discharge reaction as described in eqn (1). It should be noted that this cannot be explicitly confirmed with pressure cell data alone, as direct chemical information cannot be extracted from this approach. It is likely that two or more discharge reactions are occurring simultaneously, including the desired 2-electron ORR forming Li_2_O_2_. The overconsumption of gas is independent of whether TEMPO is present or not in the diglyme electrolyte, as demonstrated by the pressure cell measurements reported in this work in the unmediated electrolyte (Fig. S17a†), consistent with previous reports.^44^ Conversely, in the mediated sulfolane electrolyte, cell discharge proceeds more consistently close to the 2e^−^/*n*_gas_ gas consumption rate. Again, comparison to the corresponding unmediated sulfolane system (Fig. S16b and S17b†) shows that this behaviour is independent of TEMPO, suggesting that the Li[TFSI]-sulfolane electrolyte is a better medium for facilitating the 2-electron ORR than the Li[TFSI]-diglyme electrolyte under these conditions.

On charging in cycles 5–8, the instantaneous gas evolution rates in the mediated and unmediated diglyme electrolytes are consistently above the 2e^−^/*n*_gas_ rate (*i.e.*, overproduction of gas, *n*e^−^/*n*_gas_ < 2), whereas in the mediated sulfolane-based electrolyte the gas evolution rate is largely below the 2e^−^/*n*_gas_ rate (*n*e^−^/*n*_gas_ > 2), indicating that the nature of parasitic reactions is different. In the mediated diglyme electrolyte, the deviation from the ideal gas evolution rate is attributed to a decreasing capacity contribution from the TEMPO oxidation plateau and oxidation of parasitic products evolving other gases in addition to O_2_ (*e.g.*, CO_2_ from Li_2_CO_3_ oxidation).^20^ The successive loss of TEMPO functionality is even more severe in the sulfolane-based electrolytes; comparing the unmediated (Fig. S17b†) and mediated sulfolane electrolytes (Fig. 6b) shows that by cycle 6 the cell potential and instantaneous gas evolution profiles appear similar. This comparison shows that any indication of electrochemical TEMPO oxidation is lost. Therefore, Li–O_2_ cells with sulfolane-based electrolytes have a shorter lifetime than diglyme systems, exhibiting more severe polarisation on charge and capacity-fade due to build-up of parasitic products. The loss of the TEMPO oxidation plateau in the sulfolane electrolyte may be related to parasitic product accumulation blocking electrochemically active sites on the carbon electrode and/or TEMPO decomposition during cycling.

A key parasitic product that has been reported in both diglyme- and sulfolane-based electrolytes is Li_2_CO_3_.^17,39^ Bardé *et al.* suggested Li_2_CO_3_ accumulation as the cause for the rapid capacity fade in unmediated sulfolane electrolytes, as detected by infrared spectroscopy and powder X-ray diffraction on cycled carbon and nanoporous gold (NPG) positive electrodes.^39^ Despite the improved oxidative stability of NPG electrodes, Li_2_CO_3_ accumulation was still observed, highlighting electrolyte solvent decomposition as a route towards this parasitic product. Furthermore, the reactivity of Li_2_O_2_ formed may change as a function of the electrolyte; Li_2_O_2_ formed in the sulfolane-based system could be more reactive in this electrolyte environment than that formed in the diglyme system, which is consistent with the lower Li_2_O_2_ yields shown in Fig. 2 for the former. Such differences in Li_2_O_2_ reactivity depending on the electrolyte solvent have previously been reported for electrolytes based on dimethoxyethane (DME), DMSO and tetraglyme, where discharging in DMSO and tetraglyme-based systems gave lower Li_2_O_2_ yields compared to discharge in DME-based electrolytes.^56^

**Fig. 7:**
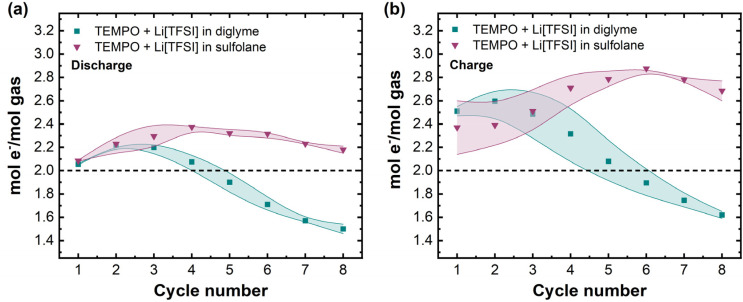
Average electron-to-gas mole ratios for mediated diglyme- and sulfolane-based electrolytes during (a) discharge and (b) charge over 8 discharge/charge cycles. Data points represent average *n*e^−^/*n*_gas_ values calculated for two cells per electrolyte and the shaded areas represent absolute uncertainty between measurements. The black dotted line shows the ideal ratio of 2e^−^/*n*_gas_.

The instantaneous gas consumption/evolution rates are useful to identify and correlate transition points in cell potential to changes in the dynamic pressure response, particularly during mediated cell charge. Importantly, such transition points cannot be discerned by considering only the average *n*e^−^/*n*_gas_ ratio per half-cycle. However, the evolution of average *n*e^−^/*n*_gas_ values over successive cycles can provide useful insight into whether the extent of parasitic chemistry grows from cycle to cycle. Fig. 7 shows the average *n*e^−^/*n*_gas_ ratio calculated for each discharge/charge half-cycle over a total of 8 cycles.

In cycle 1, in the mediated diglyme electrolyte, average *n*e^−^/*n*_gas_ = 2.06 ± 0.01, and after an initial rise, decreases to 1.50 ± 0.04e^−^/*n*_gas_. This is consistent with the overconsumption of gas observed in the instantaneous gas consumption rates (Fig. 6) for this electrolyte and shows that this overconsumption becomes more severe on cycling, *i.e.*, the continual decrease in *n*e^−^/*n*_gas_ is indicative of the extent of parasitic chemistry increasing with every cycle. In contrast, in the mediated sulfolane system, *n*e^−^/*n*_gas_ = 2.09 ± 0.01 in cycle 1, and then ranges between 2.18 ± 0.03 to 2.38 ± 0.01e^−^/*n*_gas_, indicating that while there is still a deviation from the ideal value of 2e^−^/*n*_gas_, the extent of parasitic chemistry on discharge does not grow as quickly relative to the mediated diglyme electrolyte. For the charge half-cycles, decrease in *n*e^−^/*n*_gas_ is again observed for the diglyme system, starting at 2.51 ± 0.04e^−^/*n*_gas_ and decreasing to 1.62 ± 0.03e^−^/*n*_gas_ by cycle 8, which supports previous reports of CO_2_ evolution (in addition to O_2_ evolution) from Li_2_CO_3_ oxidation in ether-based electrolytes.^17,18^ In the sulfolane electrolyte, *n*e^−^/*n*_gas_ increases from 2.37 ± 0.23e^−^/*n*_gas_ (cycle 1) to a maximum of 2.88 ± 0.01e^−^/*n*_gas_ (cycle 6). Therefore, in the mediated sulfolane system, the increasing extent of parasitic chemistry during cell charging is likely the cause for the severe capacity fade.

The pressure cell data discussed above (Fig. 5–7) demonstrates that this technique can be used to assess RM efficacy using the instantaneous gas consumption/evolution profiles. This understanding is essential given the likelihood that a practical, viable Li–O_2_ battery will require RMs for operation.^57,58^ Furthermore, the pressure cell also provides an indication of trends in parasitic chemistry over successive cycles. Further analysis was used to correlate cell potential changes with maximal gas evolution by plotting the change in cell pressure (Δ*P*) and differential capacity (d*Q*/d*V*) as functions of cell potential. This methodology is useful for close inspection to identify over what potential ranges the majority of gas evolution occurs, providing insight into the cycle-to-cycle evolution of RM activity loss.

**Fig. 8:**
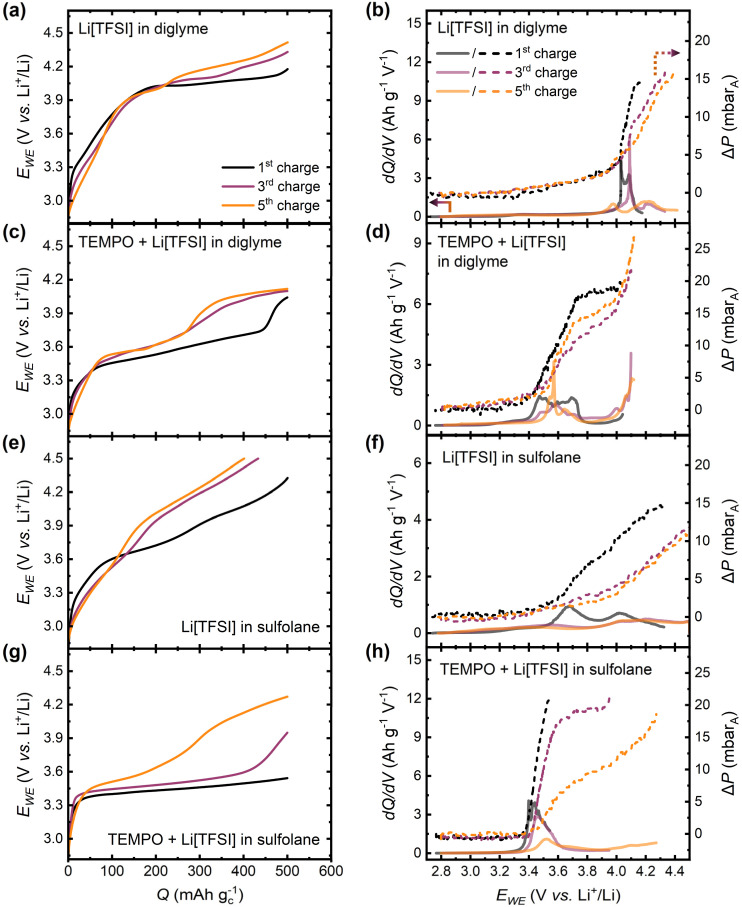
(a, c, e and g) Galvanostatic charging profiles and (b, d, f and h) differential capacity (d*Q*/d*V*, solid lines) and change in pressure (Δ*P*, dotted lines) as a function of cell potential in the 1^st^, 3^rd^ and 5^th^ charge half-cycles in (a and b) Li[TFSI]-diglyme and (c and d) TEMPO-Li[TFSI]-diglyme electrolytes, (e and f) Li[TFSI]-sulfolane and (g and h) TEMPO-Li[TFSI]-sulfolane electrolytes. The pressure at the start of each charge half-cycle is defined as Δ*P* = 0.

Fig. 8 shows d*Q*/d*V* and Δ*P* as functions of cell potential, alongside galvanostatic charging profiles, for charge half-cycles 1, 3 and 5 in mediated and unmediated diglyme and sulfolane-based electrolytes. Compared to the mediated electrolytes, the unmediated systems (Fig. 8b and f) exhibit gas evolution in cycle 1 at higher charging overpotentials and over considerably wider potential ranges. This is consistent with the mechanism of mediated *vs.* unmediated charging of Li–O_2_ cells; in the case where no TEMPO is present, Li_2_O_2_ is primarily decomposed by electrochemical oxidation, for which large overpotentials are required. In contrast, with TEMPO present (Fig. 8d and h), only electrochemical oxidation of TEMPO (occurring at lower charge overpotentials) is required to subsequently initiate chemical oxidation of Li_2_O_2_ by TEMPO^+^.

The progression of these gas evolution and d*Q*/d*V* profiles over a series of charge steps also provides another useful test for RM efficacy in Li–O_2_ cells. In cycles 3 and 5, in both mediated electrolytes, the loss in capacity of the TEMPO oxidation plateau in the galvanostatic charging profiles (Fig. 8c and g) results in gas evolution that occurs over a wider potential range (Fig. 8d and h), instead of being localised around the d*Q*/d*V* peak at the potential corresponding to TEMPO oxidation as seen in cycle 1. This is similar to what is observed in the corresponding unmediated electrolytes from the very first cycle (Fig. 8b and f), which suggests that the mechanism of charging shifts from mediated charging where Li_2_O_2_ oxidation is achieved primarily *via* chemical oxidation by TEMPO^+^, to a combination with electrochemical oxidation of Li_2_O_2_ and/or accumulated parasitic products (*e.g.*, Li_2_CO_3_). This emphasizes the importance of RMs that can also oxidise parasitic species for longer life Li–O_2_ cells.^59^ Furthermore, the loss of a distinct peak in gas evolution over cycles 1, 3, and 5 is more exacerbated in the mediated sulfolane-based electrolyte compared to the mediated diglyme-based system, consistent with the poorer rechargeability in the former upon cycling. Based on data shown in Fig. 8, some key requirements for an effective RM can be deduced. It is important that for as many charge half-cycles as possible, (i) the d*Q*/d*V* peak is centred around the RM oxidation potential (*i.e.* minimise RM oxidation plateau capacity fade) and (ii) the majority of the coupled gas evolution should be centred around the d*Q*/d*V* peak corresponding to the RM oxidation potential. Therefore, using readily accessible *operando* pressure measurements, the cell pressure change-differential capacity analysis provides a useful approach to screen RMs for the above requirements, aiding in the establishment of structure–activity relationships that are critical to the design of more effective mediators.

## Conclusion

In conclusion, *operando* pressure measurement during galvanostatic cycling of Li–O_2_ cells has been shown to be a versatile technique for assessing RM efficacy. For unexplored mediated electrolyte formulations, it is particularly useful to verify that the charging reaction proceeds *via* coupled chemical oxidation of Li_2_O_2_ (mediated by RM^+^) to evolve O_2_ gas. While this may be inferred from differences in cell potential profiles during cycling, this was confirmed during charging of Li–O_2_ cells with a TEMPO-mediated sulfolane-based electrolyte in this work. In early cycles with this electrolyte, the charge potential plateau ascribed to electrochemical TEMPO oxidation occurred at a lower potential compared to the mediated diglyme electrolyte, consistent with the redox potentials *versus* Li metal for the TEMPO^+^/TEMPO couple measured by cyclic voltammetry. Although cycling at higher O_2_ pressures improved achievable discharge capacities and rechargeability in sulfolane-based systems, capacity-fade was still more severe in this system compared to diglyme-based electrolytes. In both mediated systems, sharp rises in potential were observed during cell charging and loss of the TEMPO oxidation plateau was more rapid in the sulfolane-based electrolyte.

Interfacing a high accuracy, fast response time pressure transducer with the Li–O_2_ cell headspace enabled these rises in potential to be related to gas evolution to understand when, in a given charging step, the efficacy of the RM is diminished. The technique provides a straightforward, non-destructive/non-invasive methodology to extract the evolution of average and instantaneous *n*e^−^/*n*_gas_ ratios over many cycles, critical for a viable Li–O_2_ cell and understanding RM activity and stability. In-depth analyses demonstrated that cycle-to-cycle growth in parasitic chemistry on discharge was more severe in the mediated diglyme system, associated with an overconsumption of gas, as compared to the mediated sulfolane-based electrolyte. However, *n*e^−^/*n*_gas_ ratios on charging in the sulfolane systems deviate significantly from ideal, approaching 3e^−^/*n*_gas_, which is thought to be the primary cause for capacity fade in this electrolyte system. This was corroborated by the evolution of both differential capacity and pressure profiles as functions of the cell potential over a series of charge half-cycles. From these data, it was established that TEMPO is most effective when the majority of gas evolution is confined to a narrow potential window that centres around its redox potential in the given electrolyte. Maximising the number of cycles for which this type of response can be sustained will improve cell rechargeability and lifetime.

By combining differential capacity plots and *operando* pressure data, this analysis approach can be applied to any electrode–electrolyte combination, making it a versatile method for screening RM-containing electrolyte formulations. This is of timely importance for the Li–O_2_ battery research field, where high throughput/(semi)automated synthesis of novel RMs could be used to generate large libraries of candidate mediators, which will then require readily accessible methods for RM efficacy testing under *operando* conditions. Therefore, in the pursuit of longer-life Li–O_2_ cells, this methodology utilising on-line, highly sensitive pressure measurements in conjunction with data processing/analysis methods described herein is a valuable technique for assessing RM stability to identify and support the design of new promising RMs/electrolyte formulations.

## Author contributions

TS: conceptualisation, formal analysis, investigation, supervision, methodology, writing original draft manuscript, review and editing, and visualisation. BW: investigation, and formal analysis. ARN: conceptualisation, investigation, supervision, methodology, writing manuscript, review, and editing, and visualisation. EC: review and editing of manuscript. DJS: review and editing of manuscript. LJH: conceptualisation, funding acquisition, project administration, supervision, writing manuscript, review, and editing.

## Conflicts of interest

The authors have no conflicts of interest to declare.

## Supplementary Material

SC-016-D5SC02350E-s001

## Data Availability

The data supporting this article have been included as part of the ESI.†
