# Supplementary material for: Multicycle operando pressure measurements enable assessment of redox mediator efficacy in lithium–oxygen batteries
Source: Chem Sci. 2025 May 20;16(25):11359–74. doi: 10.1039/d5sc02350e (PMC12117508; doi:10.1039/d5sc02350e)
Supplement: SC-016-D5SC02350E-s001 [file SC-016-D5SC02350E-s001.pdf]

Supplementary Information (SI) for Chemical Science.
This journal is © The Royal Society of Chemistry 2025

Manuscript: Supplementary Information

Multicycle Operando Pressure Measurements Enable Assessment of Redox Mediator Efficacy in Lithium-Oxygen Batteries

Thukshan Samarakoon,[1] Ben Wood,[1] Alex R. Neale,[1] Elliot Coulbeck,[2] Dan Saccomando,[3] and Laurence J. Hardwick[1,*]

[1]Stephenson Institute for Renewable Energy, Department of Chemistry, University of Liverpool, Liverpool, L69 7ZF, UK

[2]Lubrizol Limited, Blackley, Manchester, M9 8ES, UK

[3]Lubrizol Limited, Hazelwood, Derby, DE56 4AN, UK

*Corresponding author: hardwick@liverpool.ac.uk

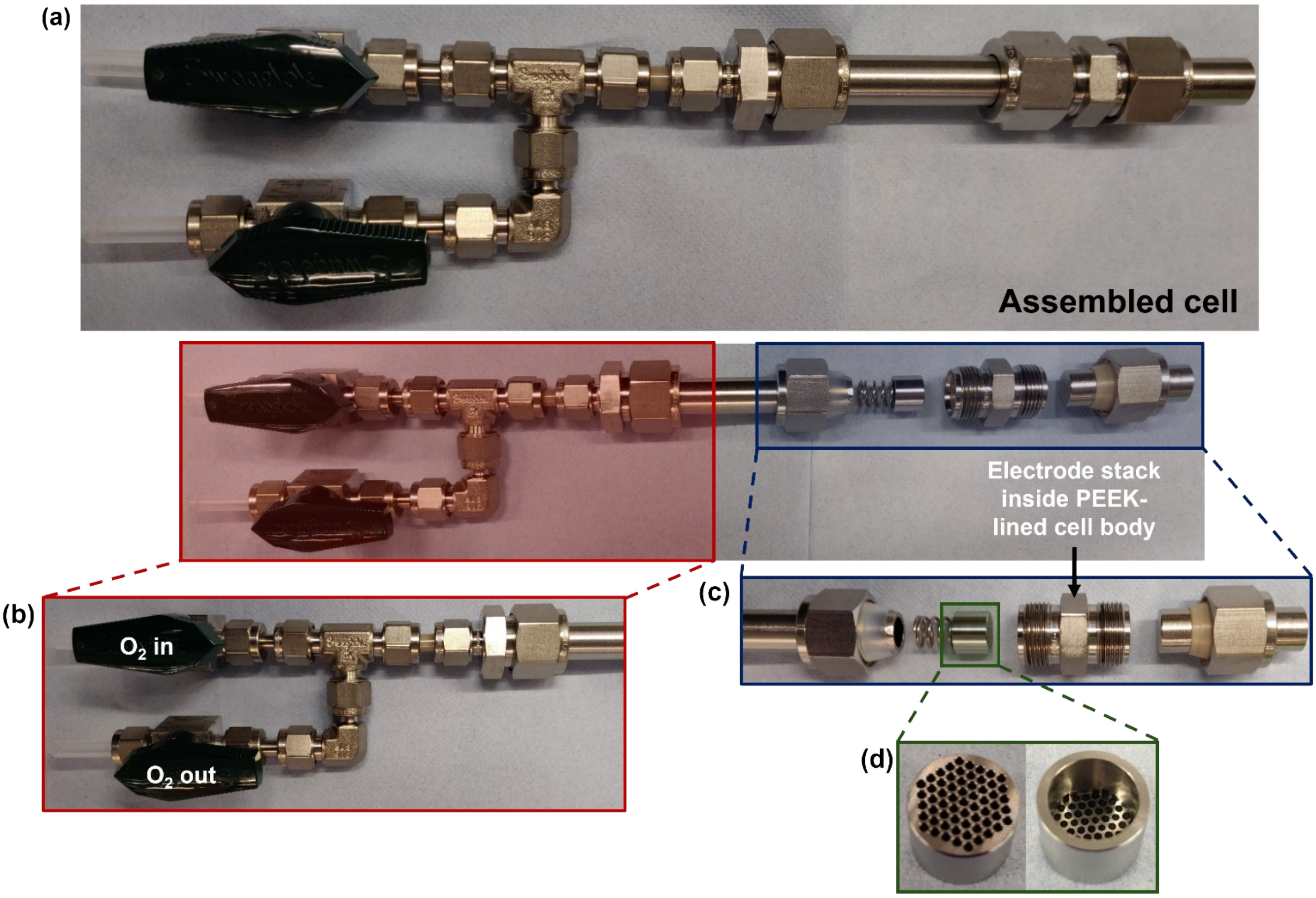

Figure S1 | A schematic of the standard Li-O_2 cell showing (a) the assembled cell, (b) quarter-turn valves with tubing to connect the cell to a high purity oxygen line for cell purging, (c) the polyether ether ketone (PEEK)-lined cell body (Microplas Mouldings Ltd.) housing the electrode stack, which is under spring-loaded compression when the cell is sealed, and (d) a stainless-steel cup with machined holes to aid uniform distribution of O_2 over the carbon electrode surface.

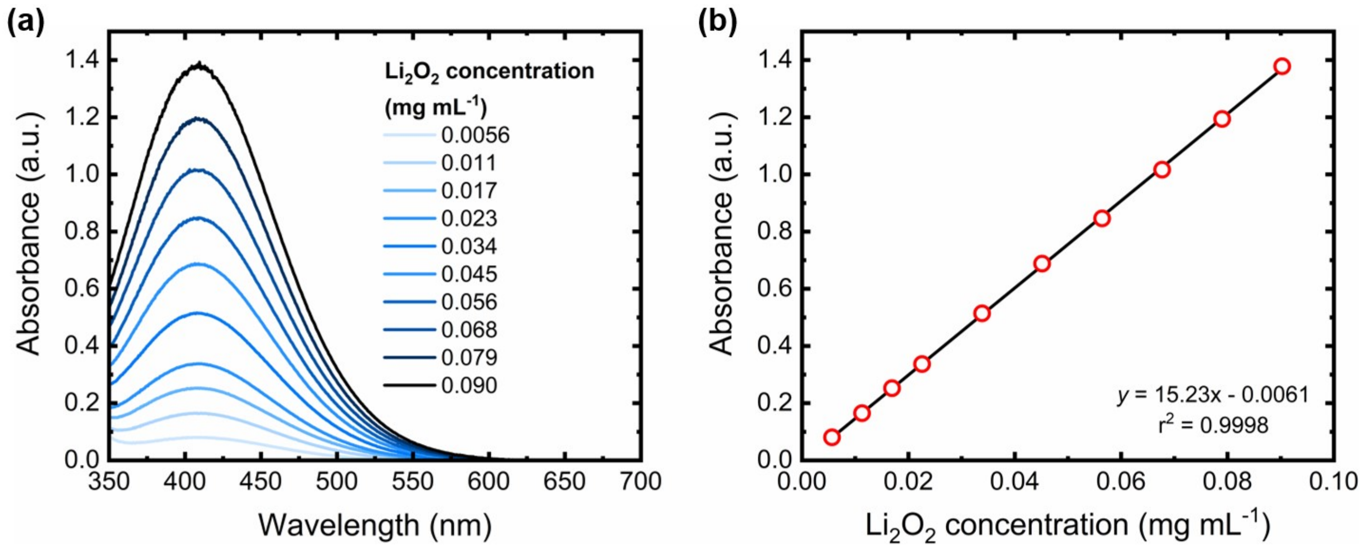

Figure S2 | (a) UV-Vis spectra of the calibration standards and **(b)** the corresponding calibration curve used for Li_2O_2 yield determination *via* UV-Vis spectroscopy. The calibration standards were prepared using known amounts of commercial Li_2O_2 (purity: 95%).

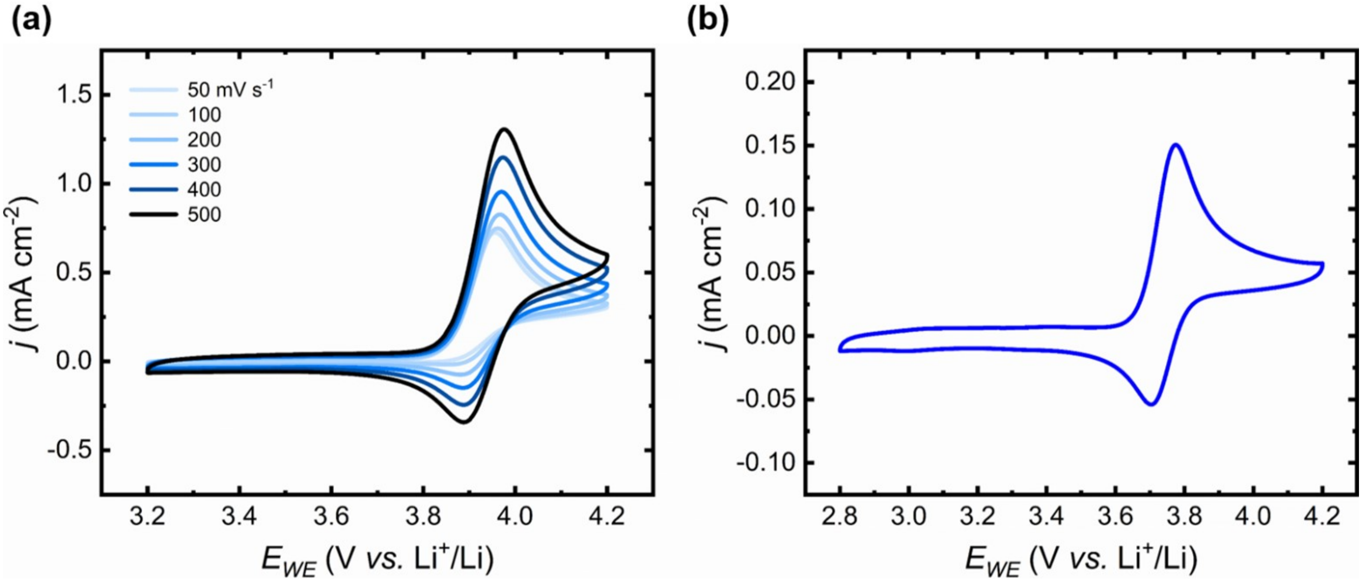

Figure S3 | Cyclic voltammograms of 10 mM TEMPO in **(a)** 1 M Li[TFSI] in DMSO at a range of scan rates (50-500 mV s^{-1}) and **(b)** 2.5 M Li[TFSI] in DMSO at 50 mV s^{-1}, highlighting partial recovery of the $TEMPO^{+}$ → TEMPO reduction peak with increasing scan rate and Li[TFSI] concentration.

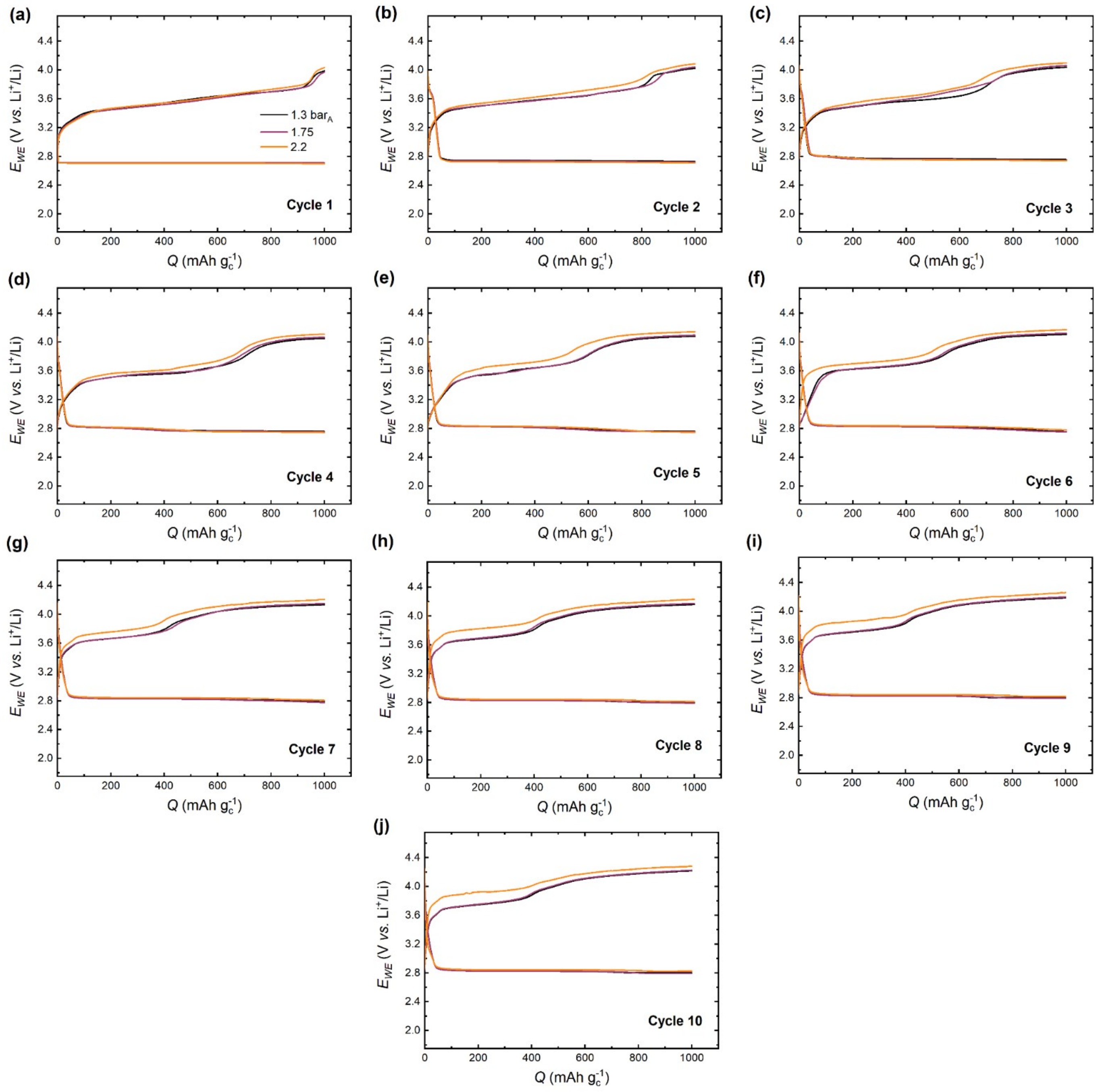

Figure S4 | Galvanostatic, capacity-limited (1000 mAh g^{-1}) cycles of Li-O_2 cells sealed at an internal O_2 gas pressure of 1.3 (black), 1.75 (purple) and 2.2 bar_A (orange) in a TEMPO-Li[TFSI]-diglyme electrolyte ($x_{solvent} : x_{Li[TFSI]} = 9 : 1$, [TEMPO] = 25 $mmol_{TEMPO}$ $kg_{solvent}^{-1}$) at 80 mA g_c^{-1}.

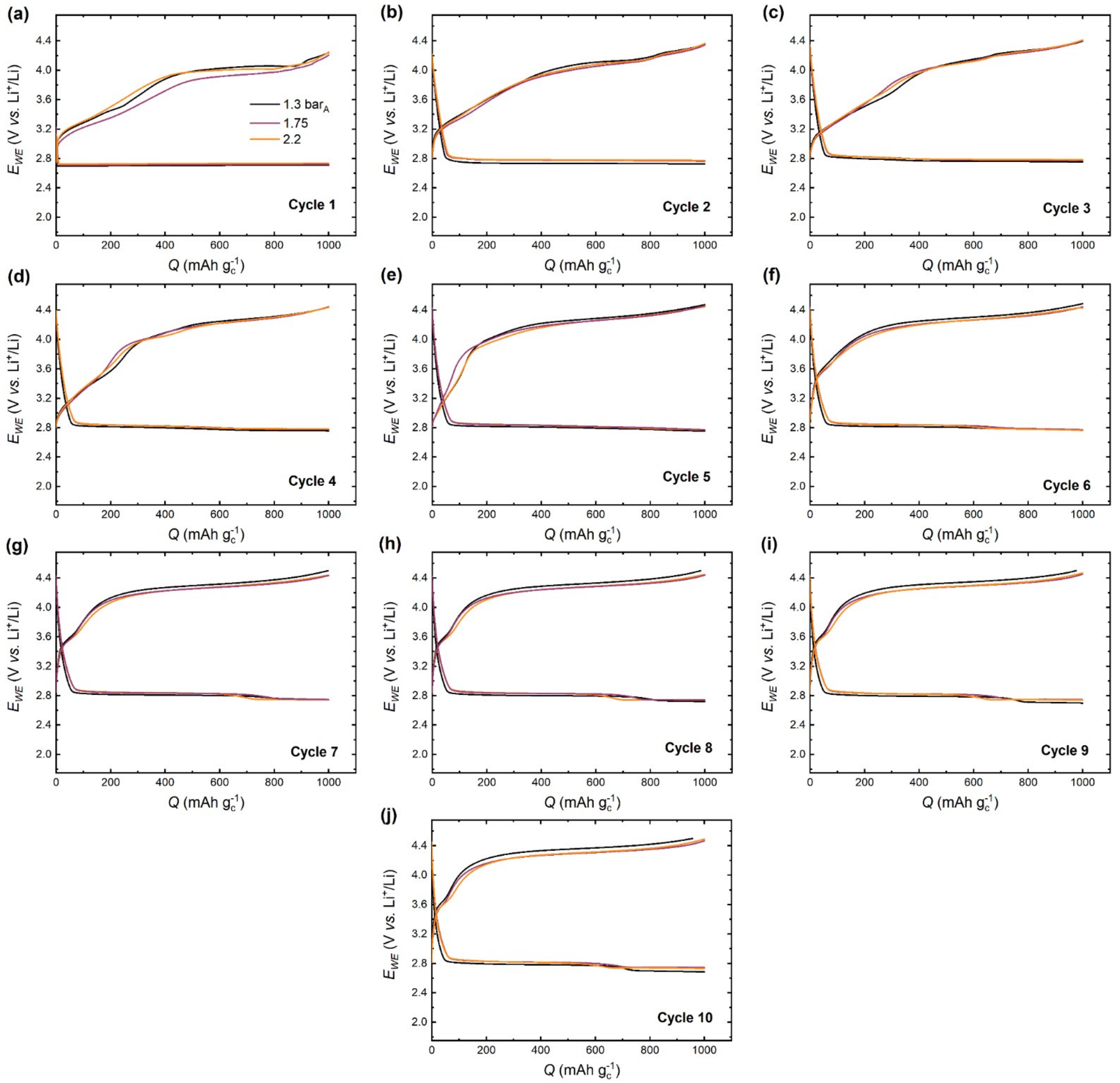

Figure S5 | Galvanostatic, capacity-limited (1000 mAh g^{-1}) cycles of Li-O_2 cells sealed at an internal O_2 gas pressure of 1.3 (black), 1.75 (purple) and 2.2 bar_A (orange) in a Li[TFSI]-diglyme electrolyte ($x_{solvent}$: $x_{Li[TFSI]}$ = 9 : 1) at 80 mA g_c^{-1}.

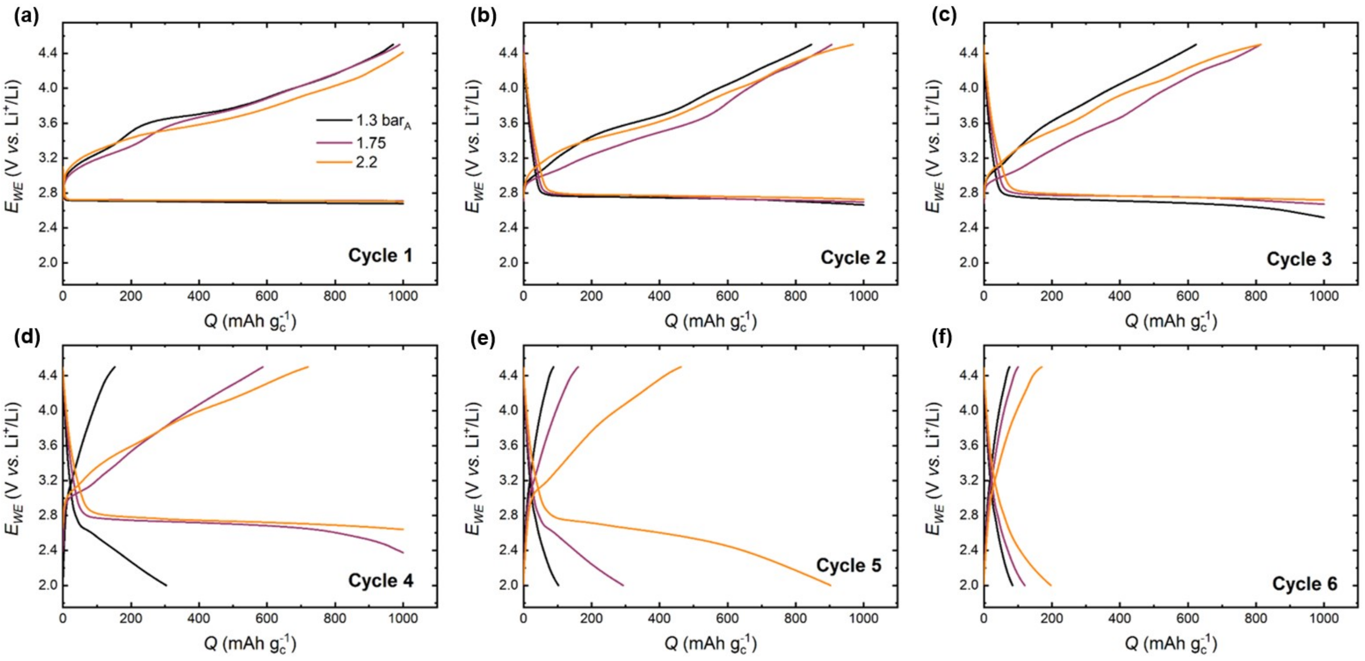

Figure S6 | Galvanostatic, capacity-limited (1000 mAh g^{-1}) cycles of Li-O_2 cells sealed at an internal O_2 gas pressure of 1.3 (black), 1.75 (purple) and 2.2 bar$_A$ (orange) in a Li[TFSI]-sulfolane electrolyte ($x_{solvent} : x_{Li[TFSI]} = 9 : 1$) at 80 mA g_c^{-1}.

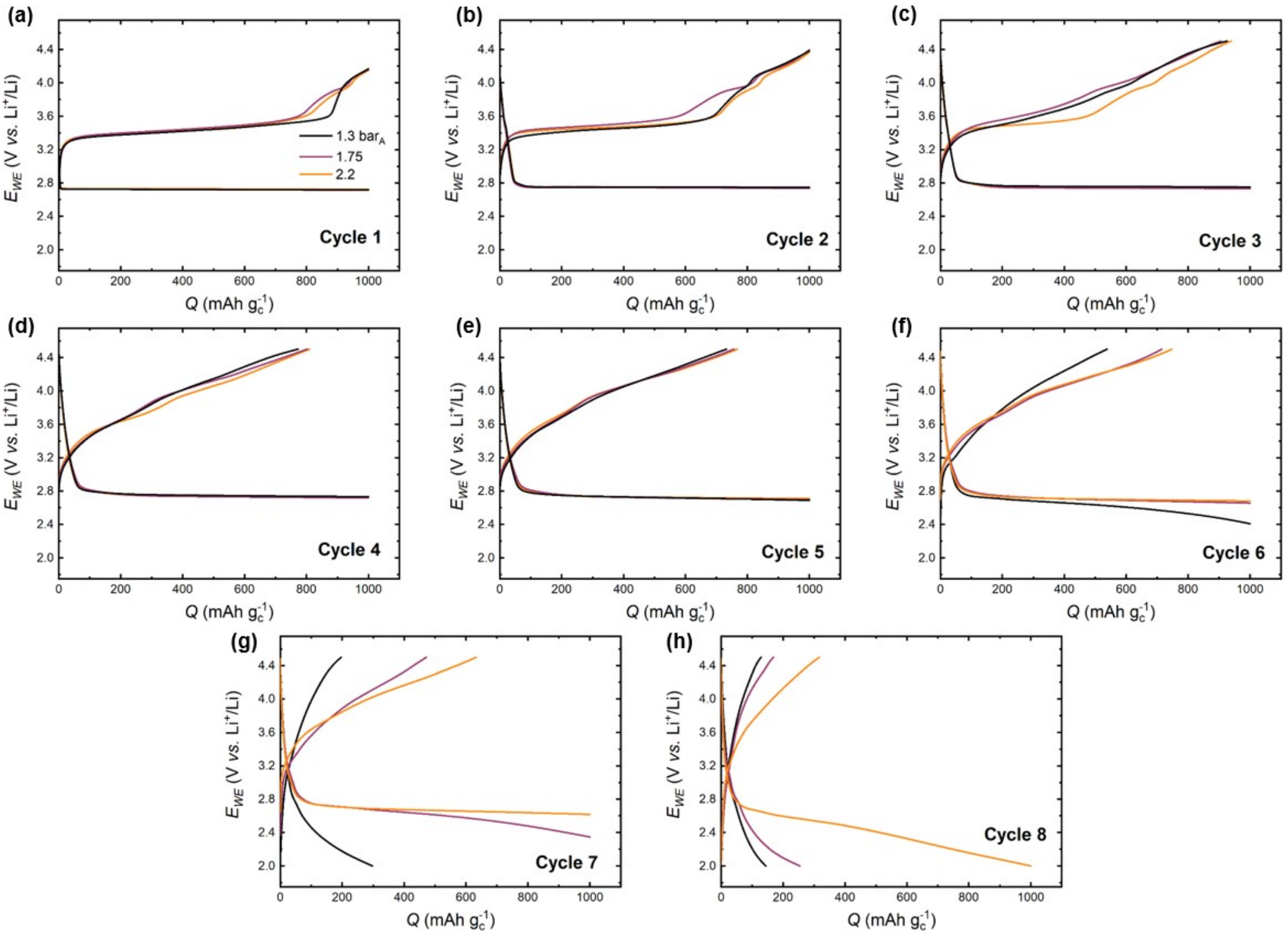

Figure S7 | Galvanostatic, capacity-limited (1000 mAh g^{-1}) cycles of Li-O_2 cells sealed at an internal O_2 gas pressure of 1.3 (black), 1.75 (purple) and 2.2 bar$_A$ (orange) in a TEMPO-Li[TFSI]-sulfolane electrolyte ($x_{solvent}$: $x_{Li[TFSI]}$ = 9 : 1, [TEMPO] = 25 mmol$_{TEMPO}$ kg$_{solvent}^{-1}$) at 80 mA g_c^{-1}.

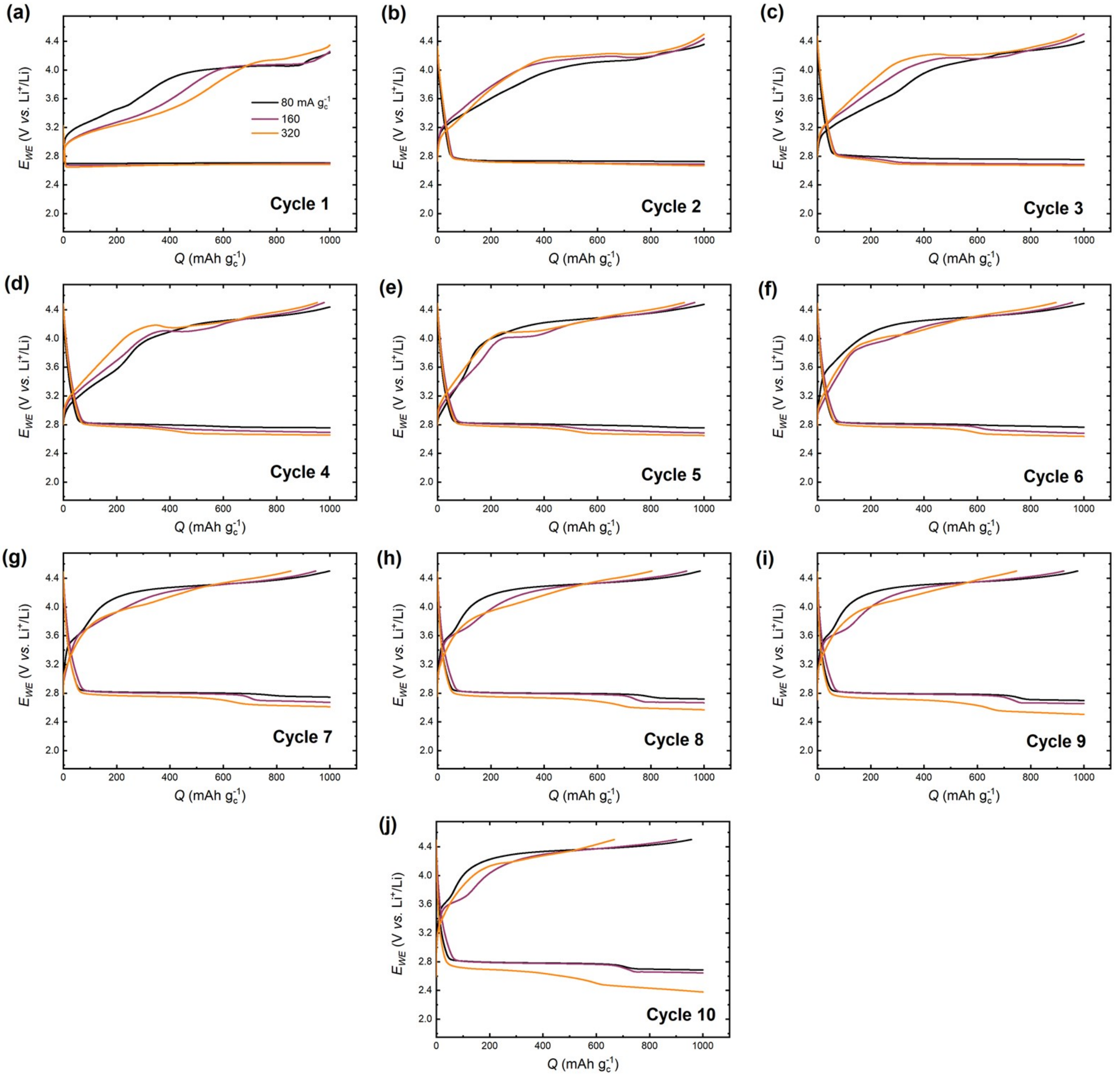

Figure S8 | Galvanostatic, capacity-limited (1000 mAh g^{-1}) cycles of Li-O_2 cells in a Li[TFSI]-diglyme electrolyte at varying current densites: 80 (black), 160 (purple) and 320 mA g_c^{-1} (orange). All cells were sealed at 1.3 bar$_A$. ($x_{solvent} : x_{Li[TFSI]} = 9 : 1$).

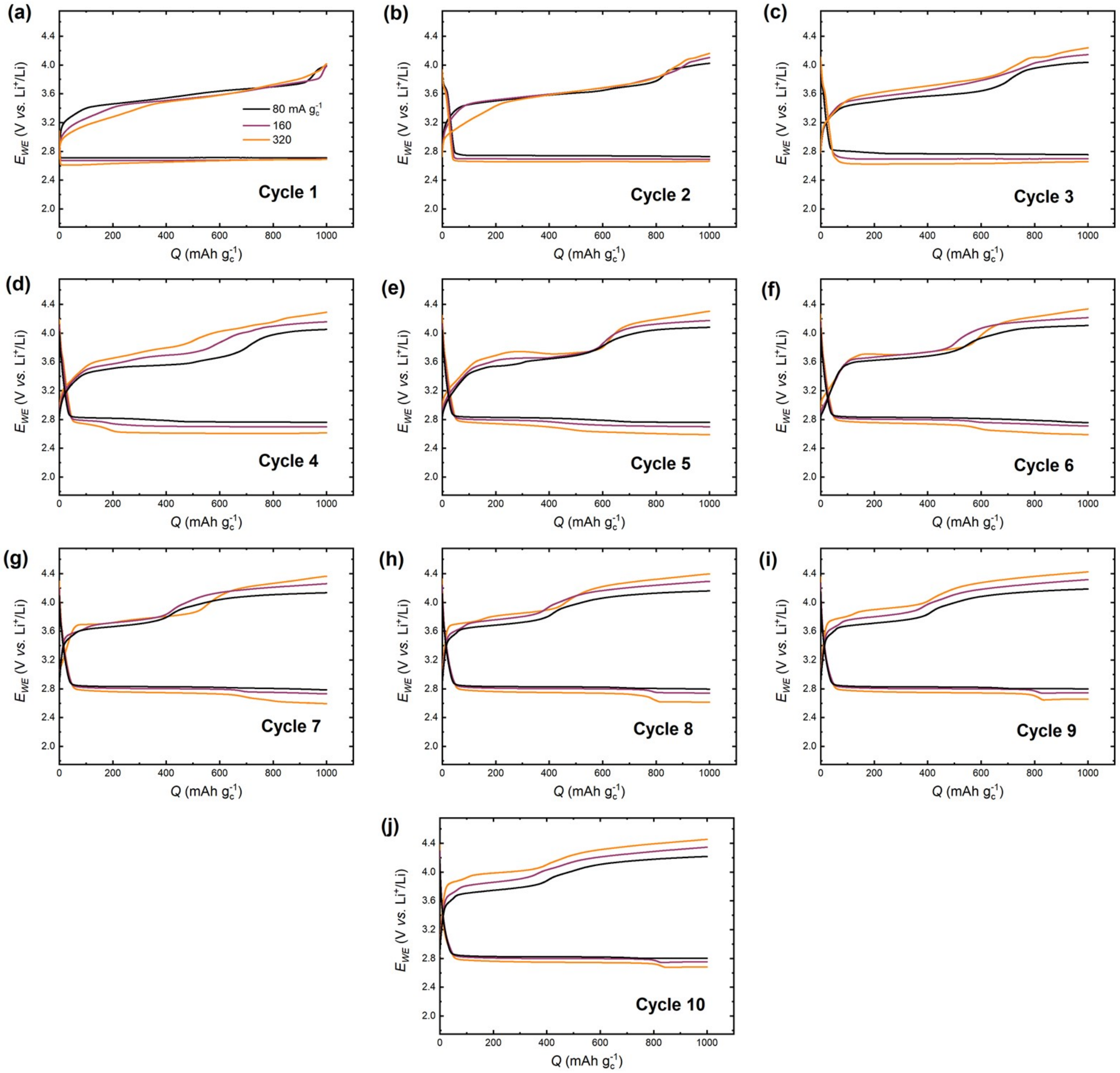

Figure S9 | Galvanostatic, capacity-limited (1000 mAh g^{-1}) cycles of Li-O_2 cells in a TEMPO-Li[TFSI]-diglyme electrolyte at varying current densites: 80 (black), 160 (purple) and 320 mA g_c^{-1} (orange). All cells were sealed at 1.3 bar_A. $x_{solvent} : x_{Li[TFSI]} = 9 : 1$, [TEMPO] = 25 $mmol_{TEMPO}\ kg_{solvent}^{-1}$.

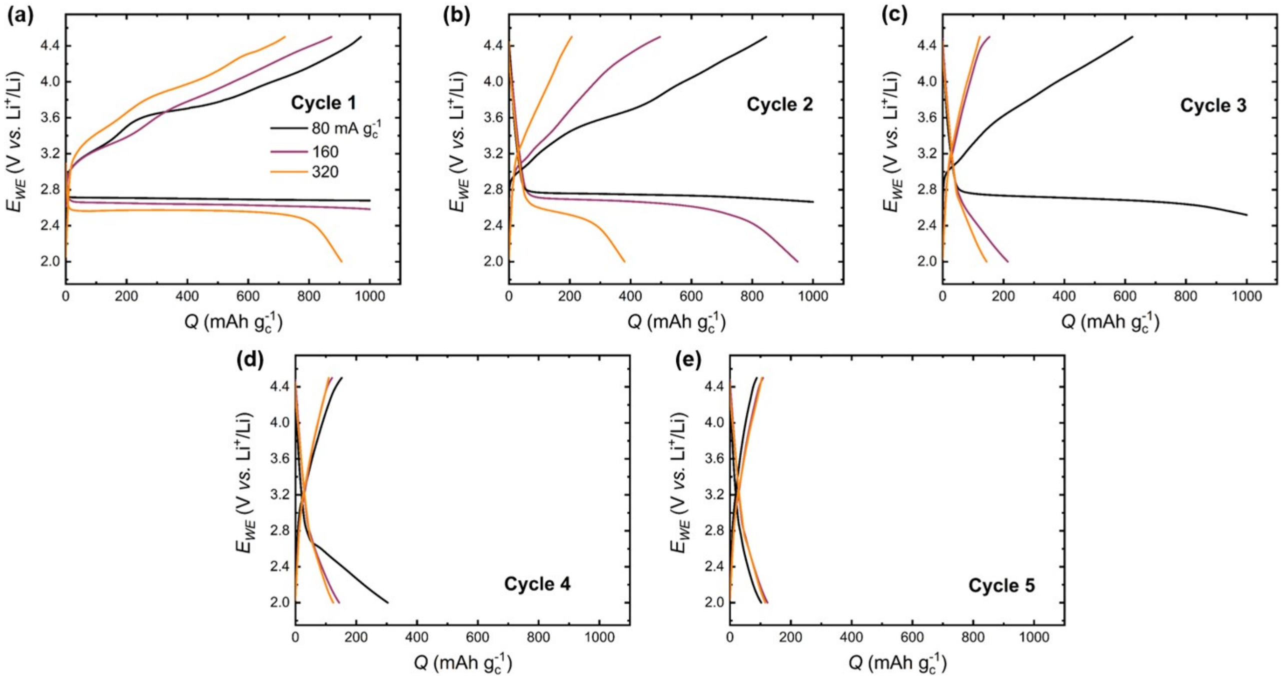

Figure S10 | Galvanostatic, capacity-limited (1000 mAh g^{-1}) cycles of Li-O_2 cells in a Li[TFSI]-sulfolane electrolyte at varying current densites: 80 (black), 160 (purple) and 320 mA g_c^{-1} (orange). All cells were sealed at 1.3 barA. $x_{solvent}$: $x_{Li[TFSI]}$ = 9 : 1.

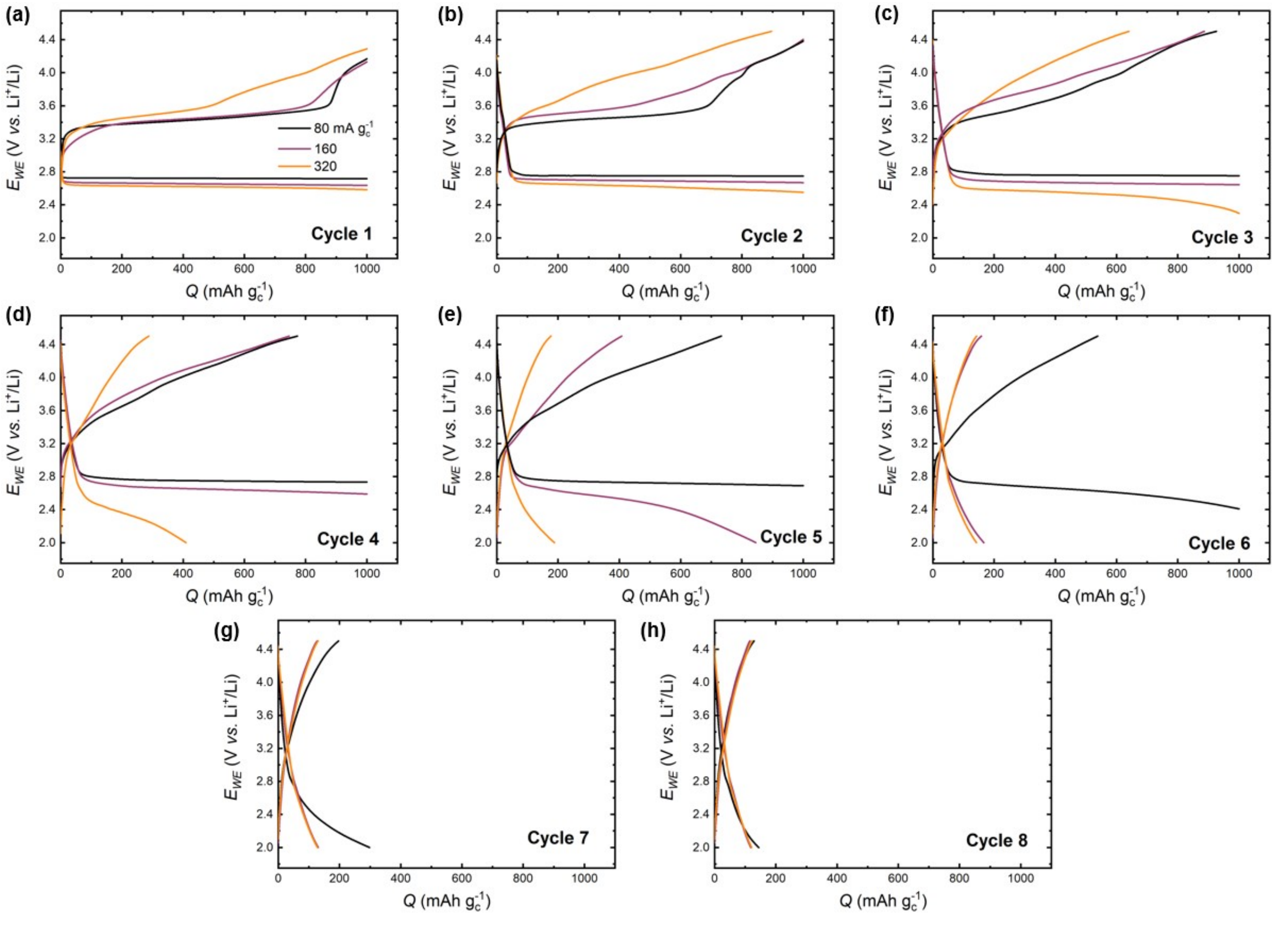

Figure S11 | Galvanostatic, capacity-limited (1000 mAh g^{-1}) cycles of Li-O_2 cells in a TEMPO-Li[TFSI]-sulfolane electrolyte at varying current densites: 80 (black), 160 (purple) and 320 mA g_c^{-1} (orange). All cells were sealed at 1.3 bar_A.$x_{solvent} : x_{Li[TFSI]} = 9 : 1$, [TEMPO] = 25 $mmol_{TEMPO}$ $kg_{solvent}^{-1}$.

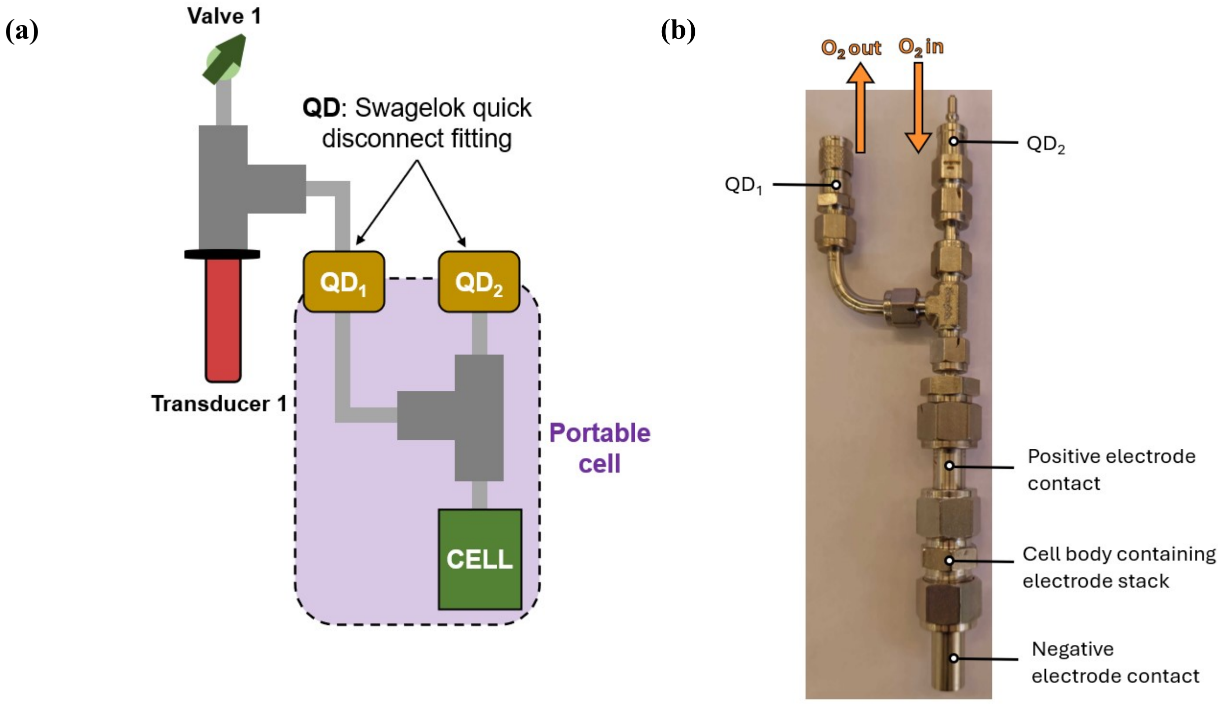

Figure S12 | **(a)** Schematic of the operando pressure-electrochemical cell connected to a pressure transducer *via* a quick disconnect fitting (QD_1) highlighting the portable cell portion. **(b)** An image of the portable cell showing the direction of O_2 flow into and out of the cell. The cell body containing the electrode stack has an internal lining made of polyetheretherketone (PEEK) to prevent short circuiting. The cell headspace is the volume between QD_2 and valve 1.

A schematic of the operando pressure-electrochemical cell is shown in **Figure S12**. The purple shaded region labelled ‘‘Portable cell’’ shows the portion of the setup that can be disconnected at QD_1 and taken into the glovebox for cell

assembly and sealing. The green box labelled ‘‘CELL’’ contains the electrode stack (stainless steel mesh, carbon black positive electrode, glass fibre separator soaked with electrolyte and Li metal negative electrode). Prior to cycling, the portable cell was connected to pressure transducer 1 *via* QD_1, and then purged by connecting a high purity O_2 gas line to QD_2 and opening valve 1. Following a 30 min purge step, the system was sealed with a defined pressure of gas in the cell headspace (volume between QD_2 and valve 1), rested for 18-24 h to achieve a stable leak rate, and then galvanostatically cycled.

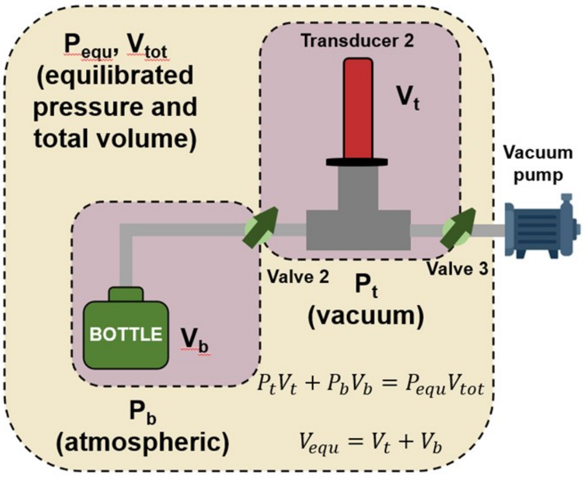

Figure S13 | A schematic illustrating how the volume of transducer 2 was determined using a stainless-steel bottle of known volume.

To determine the volume of the cell headspace (all components between valve 1 and QD_2 in its closed position) as shown in **Figure S12**, a second pressure transducer of known volume was required. Therefore, a stainless-steel bottle of known volume was connected to transducer 2 as shown in **Figure S13**. The purple shaded region of volume V_b was determined by the difference in mass between the dry bottle and the bottle filled with deionised water (taking into account volume of fittings required to connect the bottle to valve 2). This was done multiple times to determine V_b = 11.25 ± 0.02 mL.

To determine the volume V_t, valves 2 and 3 were closed and opened, respectively, and a partial vacuum (no more than 0.3 bar_A) was pulled in volume V_t. Valve 3 was closed, keeping V_t at reduced pressure P_t, while V_b remained at atmospheric pressure P_b. Once P_t had stabilised, valve 2 was opened, bringing the system with total volume V_{tot} to an equilibrated pressure P_{equ}. Using P_{equ} and the two equations shown in **Figure S13**, it was calculated that V_t = 4.33 ± 0.01 mL.

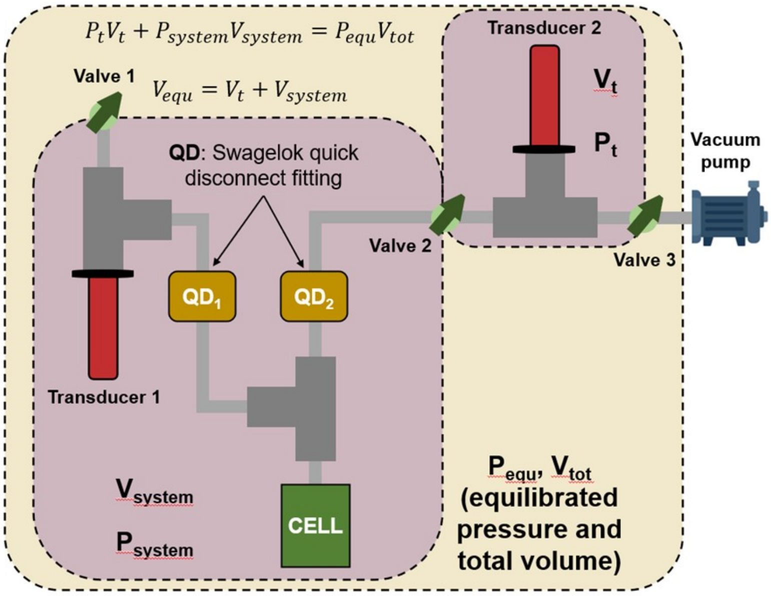

Figure S14 | A schematic of the pressure cell with a second pressure transducer of known volume, connected *via* a quick disconnect fitting (QD_2).

The internal cell volume denoted by V_{system} (*i.e.* from valve 1 to QD_2) can be determined in a similar way as described above for the calculation of V_t. As shown in **Figure S14**, V_{system} is the internal cell volume of interest, which was used to determine ideal gas evolution rates and electron-to-gas mole ratios (n_e/n_{gas}). To determine V_{system}, volume V_t was evacuated by opening valve 3 while keep valve 2 closed. After closing valve 3, V_t was under reduced pressure, while V_{system} was at atmospheric pressure P_{system}. Valve 2 was then opened to equilibrate the entire setup represented by the beige shaded region, which is now has a total volume V_{tot} and is at pressure P_{equ}. Then, as was done for determination of V_t, using P_{equ} and the two equations shown in **Figure S14**, the system volume was calculated as $V_{system} = 12.06 \pm 0.01$ mL. This value takes into account the volume of the electrode stack (a dry cell containing all stack components was assembled for these measurements), and is arrived at by subtracting the volume contributions stemming from the electrolyte added (0.080 mL) and the stainless-steel tubing used to connect QD_2 to valve 2 (0.36 mL). In the determination of V_{system} as described above, QD_2 is in the open configuration, but when cycling the pressure cell, QD_2 is in the closed position. However, given the negligible difference in internal volume of the quick disconnect fittings in their open and closed configurations, the associated volume changes were not considered in the calculation of V_{system}.

<u>Supplementary Note 1 – Processing operando pressure cell data</u>

Raw pressure profiles in the pressure cell data were smoothed using a locally estimated scatterplot smoothing function in Origin graphing software and corrected for the leak rate determined during the rest period (**Figure S15**). The instantaneous pressure profiles (dP/dt) were smoothed using a percentile filter function in Origin.

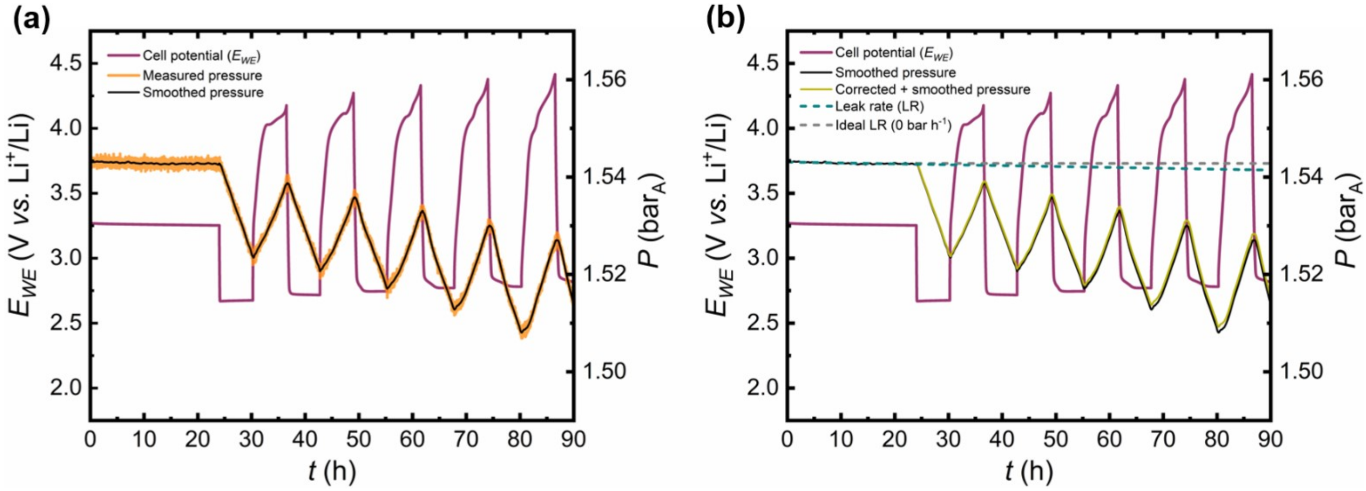

Figure S15 | Example graphs to illustrate typical data processing procedure for pressure cell data (electrolyte: Li[TFSI]-diglyme). The cell potential (purple), smoothed pressure (black) and leak rate (cyan, dotted) are shown as a function of time with **(a)** the measured (raw) pressure reading (orange) and **(b)** the corrected and smoothed pressure (olive) with ideal leak rate (grey, dotted). The ideal leak rate assumes no leaking and is fixed at the pressure at the start of discharge. The leak rate in this example during the 24 h open circuit (rest) period was 1.8 x 10^{-5} bar h^{-1}.

Supplementary Note 2 – Example calculation of average electron-to-gas mole (n_e/n_{gas}) ratio

1st discharge half-cycle in unmediated diglyme electrolyte

Terms: moles (n), cell volume (V), universal gas constant (R), temperature (T), capacity (Q),pressure (P).

$Pressure\ at\ start\ of\ 1st\ charge\ =\ 1.515\ bar$
$Pressure\ at\ end\ of\ 1st\ charge =\ 1.496\ bar$
$Pressure\ difference = 0.0191\ bar = 1911\ Pa$

$Cell\ volume = 12.06\ mL = 1.206\ \times 10^{-5}\ m^3$

$$n_{gas} = \frac{PV}{RT} = \frac{(1911\ Pa \times 1.206 \times 10^{-5}\ m^3)}{(8.3145\ J\ K^{-1}\ mol^{-1} \times 303\ K)} = 9.149 \times 10^{-6}\ mol = 9.15\ \mu mol$$

Assuming that all gas consumed is oxygen ($n_{gas} = n_{O_2}$), then, in theory, 2 moles of charge should be passed for every mole of gas consumed based on a 2-electron oxygen reduction reaction ($O_2 + 2\ Li^+ + 2\ e^- \rightarrow Li_2O_2$).

$n_{e,\ theoretical} = 2n_{O_2} = 18.30\ \mu mol$

The charge passed (in mAh) based on a 500 mAh g^{-1} discharge/charge capacity limit and a carbon black loading for this particular cell of 1.0 mg is 0.50 mAh.

$$Q = \frac{0.50\ mAh \times 3600\ s\ h^{-1}}{1000} = 1.81\ C$$

$$n_{e,\ experimental} = \frac{1.81\ C}{96485\ C\ mol^{-1}} = 1.875 \times 10^{-5}\ mol = 18.75\ \mu mol$$

Therefore, the average n_e/n_{gas} for this charge half-cycle is calculated as:

$$\frac{n_{e,\ experimental}}{n_{O_2}} = \frac{18.75}{9.15} = 2.05\ e^-/O_2$$

Supplementary Note 3 – Example calculation of instantaneous gas consumption/evolution rates

Unmediated diglyme electrolyte

The instantaneous gas consumption/evolution rates were calculated by taking the first derivative of the pressure response.

An example calculation of theoretical gas consumption/evolution rates is shown below for a cell containing the unmediated diglyme electrolyte (same cell as in Supplementary Note 2).

The charge passed (in mAh) based on a 500 mAh g^{-1} discharge/charge capacity limit and a carbon black loading for this particular cell of 1.0 mg is 0.50 mAh. Again, assuming that all gas consumed is oxygen ($n_{gas} = n_{O_2}$), then, in theory, 2 moles of charge should be passed for every mole of gas consumed based on a 2-electron oxygen reduction reaction ($O_2 + 2\ Li^+ + 2\ e^- \rightarrow Li_2O_2$). Therefore, as shown in Supplementary Note 2:

$$n_{e,\ experimental} = 18.75\ \mu mol$$

$$n_{gas,\ theoretical} = n_{O_2,\ theoretical} = \frac{n_{e,\ experimental}}{2} = 9.38\ \mu mol$$

The time (t) for single discharge/charge half cycle where the above capacity limit (0.50 mAh) is reached and a specific current of 80 mA g^{-1} (= 80.427 µA) is applied is given by:

$$t = \frac{0.50\ mAh}{0.080427\ mA} = 6.25\ h$$

The theoretical average gas consumption rate (in µmol h^{-1}) over this period assuming a 2 e^-/n_{gas} process:

$$\frac{n_{O_2,theoretical}}{t} = \frac{9.38\ \mu mol}{6.25\ h} = 1.50\ \mu mol\ h^{-1}$$

The theoretical average gas consumption rate (in mbar h^{-1}) over this period assuming a 2 e^-/n_{gas} process:

$$P_{theoretical} = \frac{n_{O_2,theoretical}RT}{V} = \frac{9.38 * 10^{-6}\ mol \times 8.3145\ J\ K^{-1}mol^{-1} \times 303\ K}{1.206 \times 10^{-5}\ m^3} = 1959\ Pa = 0.0196\ bar$$

$$\frac{dP_{theoretical}}{dt} = \frac{0.0196\ bar}{6.25\ h} = 0.00313\ bar\ h^{-1} = 3.13\ mbar\ h^{-1}$$

The same procedure was followed to calculate the theoretical average gas consumption/evolution rate for a 4 e^-/n_{gas} process with:

$$n_{gas,\ theoretical} = n_{O_2,\ theoretical} = \frac{n_{e,\ experimental}}{4} = \frac{18.75\ \mu mol}{4} = 4.69\ \mu mol$$

$$\frac{n_{O_2,theoretical}}{t} = \frac{4.69\ \mu mol}{6.25\ h} = 0.75\ \mu mol\ h^{-1}$$

$$P_{theoretical} = \frac{n_{O_2,theoretical}RT}{V} = \frac{4.69 * 10^{-6}\ mol \times 8.3145\ J\ K^{-1}mol^{-1} \times 303\ K}{1.206 \times 10^{-5}\ m^3} = 980\ Pa = 0.00980\ bar$$

$$\frac{dP_{theoretical}}{dt} = \frac{0.00980\ bar}{6.25\ h} = 0.00157\ bar\ h^{-1} = 1.57\ mbar\ h^{-1}$$

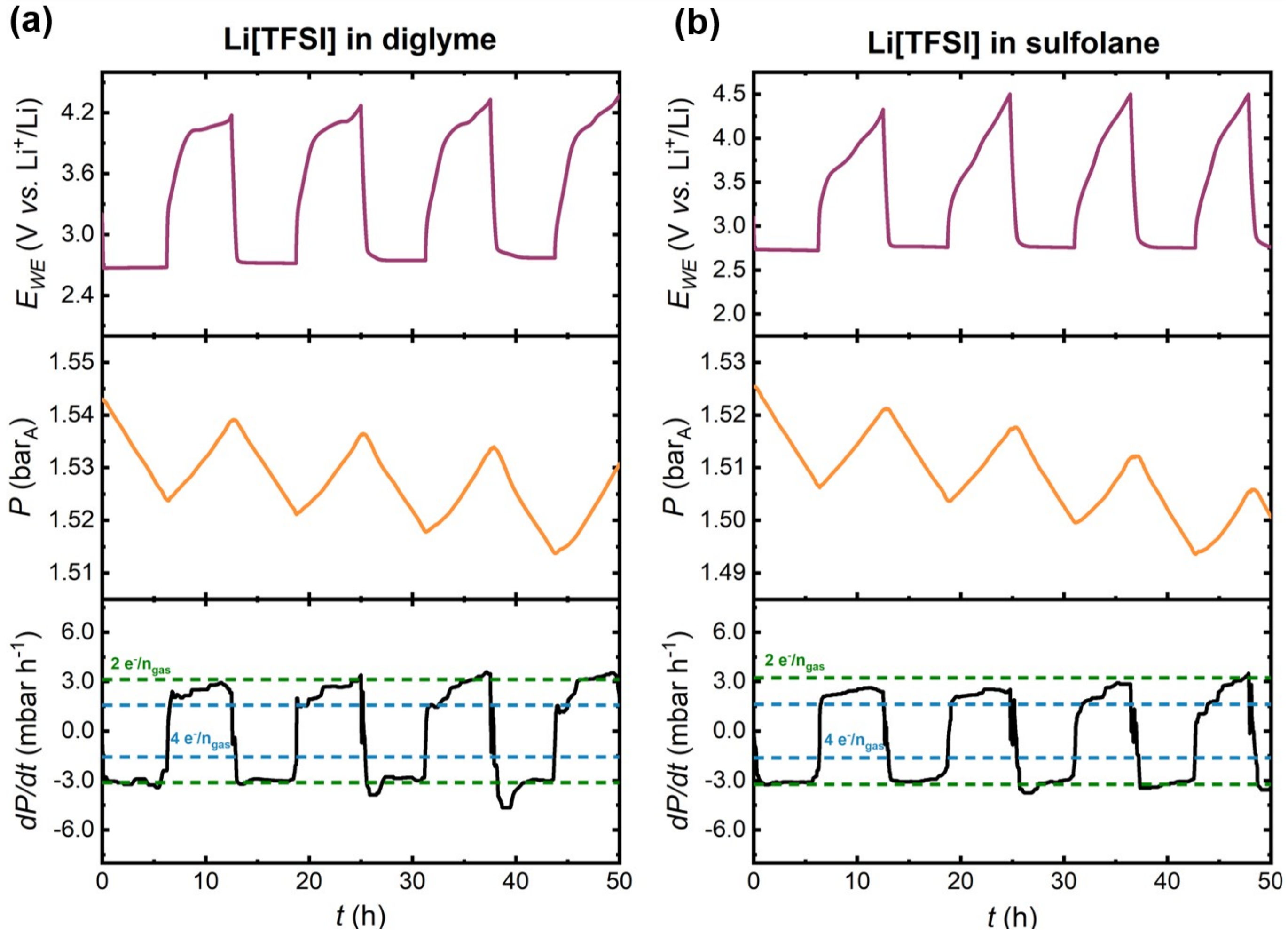

Figure S16 | Cycles 1-4 in the pressure cell cycled under a capacity-limited (500 mAh g^{-1}) regime at 80 mA g^{-1} in unmediated **(a)** diglyme-and **(b)** sulfolane-based electrolytes. The mole fraction (*x*) ratio of solvent : Li[TFSI] salt was $x_{\mathrm{solvent}} : x_{\mathrm{Li[TFSI]}}$ = 9 : 1. The cell potential, pressure response and 1st derivative of the pressure response are shown as a function of time. The dotted green and blue lines indicate the theoretical gas consumption/evolution rates for 2 e$^-$/n$_{gas}$ and 4 e$^-$/n$_{gas}$ processes, respectively.

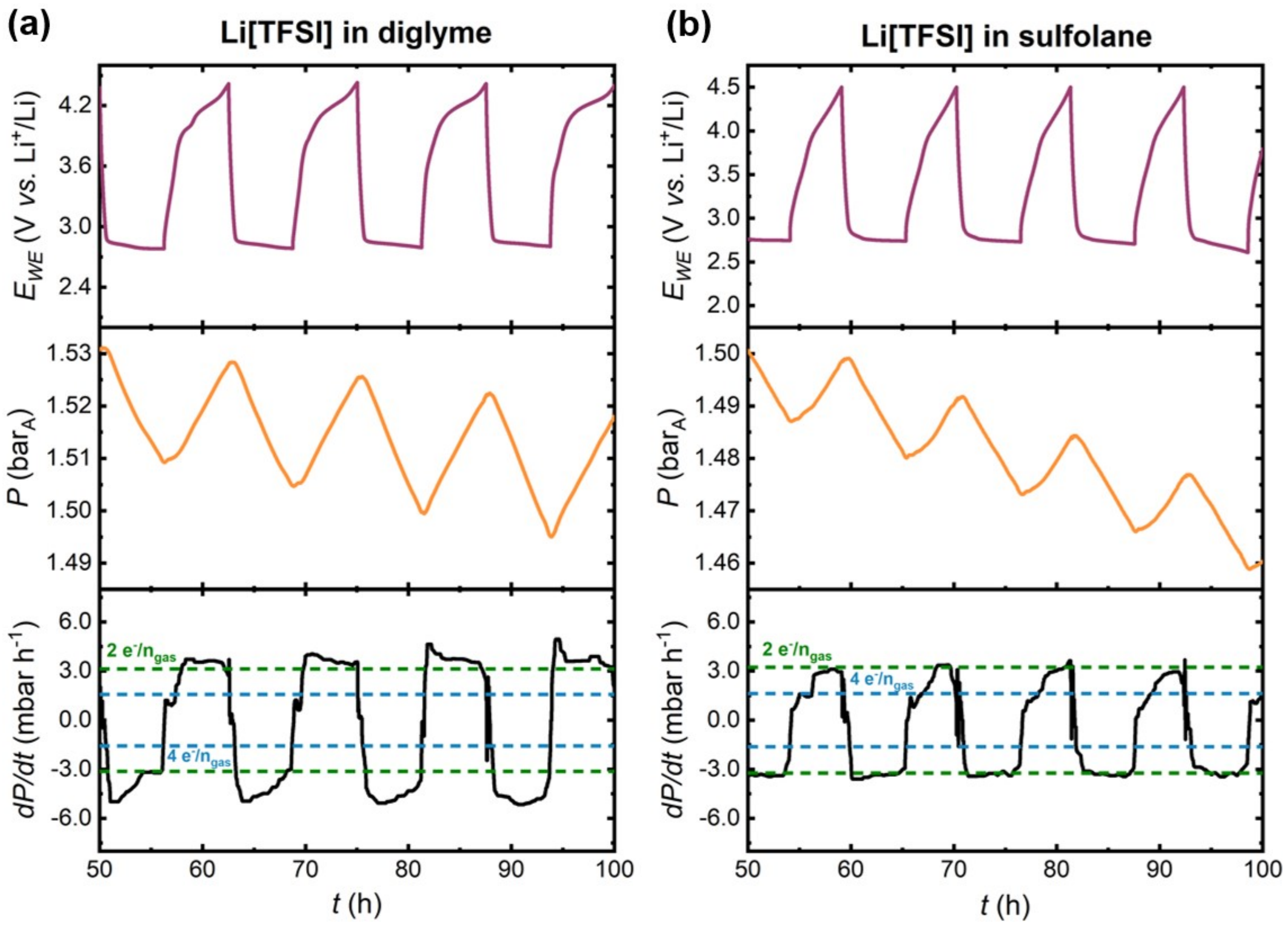

Figure S17 | Cycles 5-8 in the pressure cell cycled under a capacity-limited (500 mAh g^{-1}) regime at 80 mA g^{-1} in unmediated **(a)** diglyme-and **(b)** sulfolane-based electrolytes. The mole fraction (x) ratio of solvent : Li[TFSI] salt was $x_{solvent} : x_{Li[TFSI]}$ = 9 : 1. The cell potential, pressure response and 1st derivative of the pressure response are shown as a function of time. The dotted green and blue lines indicate the theoretical gas consumption/evolution rates for 2 e^-/n_{gas} and 4 e^-/n_{gas} processes, respectively.
